# Bioactive Ingredients, Functions, and Development Strategies of *Phascolosoma esculenta*—An Edible Marine Organism: A Review

**DOI:** 10.1002/fsn3.70217

**Published:** 2025-05-08

**Authors:** Lingxuan Chen, Wen Xu, Rui Zhu, Shaohua Xu, Lixia Chen, Hua Li

**Affiliations:** ^1^ Institute of Structural Pharmacology & TCM Chemical Biology, Fujian Key Laboratory of Chinese Materia Medica, College of Pharmacy Fujian University of Traditional Chinese Medicine Fuzhou China; ^2^ Wuya College of Innovation, School of Pharmacy, Key Laboratory of Structure‐Based Drug Design & Discovery, Ministry of Education Shenyang Pharmaceutical University Shenyang China

**Keywords:** adaptability, bioactive ingredients, development strategies, *Phascolosoma esculenta*, physiological functions

## Abstract

Marine organisms represent a significant source for foods and medicines. *Phascolosoma esculenta* as an edible marine organism grows in the intertidal zone along the southern coast of China. It is high in protein and low in fat with excellent nutritional value. Various studies have shown that *P. esculenta* contains numerous bioactive ingredients with potential physiological functions, including anti‐inflammatory, antioxidant, liver and cardiovascular protective, cerebrovascular protective, and immune‐regulating properties. Moreover, *P. esculenta* possesses a range of antioxidant proteins that mitigate oxidative damage resulting from environmental stress, making it a candidate for use in environmental monitoring. Therefore, it holds significant potential across various sectors, including food, medicine, nutrition and health care, and environmental monitoring. This paper concludes by summarizing the bioactive ingredients and functions of *P. esculenta* as well as various research technologies related to marine functional foods, aiming to provide a foundation for developing *P. esculenta* into green foods or medicinal products.

## Graphical abstract

*Phascolosoma esculenta* is an invertebrate living in the ocean's intertidal zone, belonging to the phylum Sipuncula, and is often cooked as a delicious snack in China's coastal areas. It is often referred to as the “sea ginseng” because of its high nutritional value and the efficacy of enhancing endurance. This paper reviews the active ingredients and pharmacological effects of *P. esculenta* , summarizes its tolerance, and lays the foundation for future extensive artificial breeding efforts. Additionally, various techniques for the development of marine food are compiled with the aim of further exploring *P. esculenta* as a potential healthcare food.

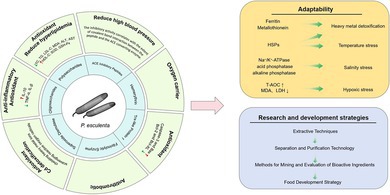

**Abbreviations**
ACE: angiotensin I‐converting enzyme
ALT: alanine aminotransferase
AST: aspartate aminotransferase
BMSCs: bone marrow mesenchymal stem cells
Cu/ZnSOD: copper‐–zinc superoxide dismutase
DPPH: 2,2‐diphenyl‐1‐picrylhydrazyl
FT‐IR: fFourier‐transform infrared
GSH‐Px: glutathione peroxidase
HDL‐C: high‐density lipoprotein cholesterol
HSP: heat shock proteins
IL‐10: interleukin‐10
IL‐1β: interleukin‐1beta
LDL‐C: low‐density lipoprotein cholesterol
MDA: malondialdehyde
MnSOD: manganese superoxide dismutase
PeFer:
*Phascolosoma esculenta* ferritin
ROS: reactive oxygen species
SOD: superoxide dismutase
TC: cholesterol
TG: triglycerides
TNF‐α: tumor necrosis factor‐alpha
Trx: thioredoxin

## 1 Introduction

The ocean, constituting approximately 70% of the earth's surface, serves as an immense reservoir of species diversity and abundant resources. The ocean's conditions of high pressure, salinity, low oxygen, and limited light have fostered distinct metabolic, adaptive, and compositional traits in marine organisms, distinguishing them from terrestrial counterparts. Metabolites from marine organisms with unique chemical structures and enzyme reaction mechanisms have become a source for the development of new drugs or functional foods. For example, fish oil derived from deep‐sea fish is rich in omega‐3, which has been proved to have significant anti‐inflammatory effects. It is commonly utilized as a dietary supplement to mitigate cardiovascular and cerebrovascular ailments, as well as an adjunct therapy for hyperlipidemia and hypertension (Shahidi and Ambigaipalan 2018). Marine invertebrates are rich in biologically active secondary metabolites, several of which have been developed as drugs, such as vidarabine (an antiviral drug isolated from sponges), cytarabine (an antileukemia drug isolated from sponges), and trabectedin (an anticancer drug isolated from the *Caribbean sheath*; Buchanan and Hess 1980; Glantz et al. 1999; Wang et al. 2022). As of the conclusion of 2016, more than 218,500 marine‐derived natural products have been cataloged (Haque et al. 2022). Many of these are in various stages of clinical research for drug development. Table 1 provides an overview of marine‐derived pharmaceuticals marketed globally. Nevertheless, there are still huge marine resources that deserve further development.

**TABLE 1:** Marketed drugs of marine and their therapeutic applications.

Source		Medicines name	Treatment of disease	Approved listing organizations and years
Porifera	*Cryptotethia crypta*	Cytarabine, Ara‐C	Acute nonlymphocytic leukemia; Lymphomatous meningitis	FDA (1969)
	*Cryptotethia crypta*	Vidarabine, Ara‐A	Viral ophthalmopathy, herpes zoster, herpes simplex conjunctivitis	FDA (1976)
	*Cryptotethia crypta*	Fludarabine, F‐ara‐A	B‐cell chronic lymphocytic leukemia	FDA (1991)
	*Cryptotethia crypta*	Nelarabine	T‐cell acute lymphoblastic leukemia and T‐cell lymphoblastic lymphoma	FDA (2005)
	*Halichondria okadai*	Eribulin mesylate	Metastatic breast cancer; unresectable or metastatic soft tissue sarcoma	FDA (2010)
Mollusca	*Dolabella auricularia*	Brentuximab vedotin	Adult CD30‐positive relapsed or refractory systemic anaplastic large cell lymphoma and classical Hodgkin ‘s lymphoma	FDA (2019)
	*Dolabella auricularia*	Polatuzumab vedotin	Recurrent and refractory diffuse large B‐cell lymphoma	FDA (2019)
	*Dolabella auricularia*	Enfortumab vedotin	Patients with locally advanced or metastatic urothelial carcinoma	FDA (2019)
	*Dolabella auricularia*	Belantamab mafodotin	Recurrent or refractory multiple myeloma	FDA (2020)
	*Conus magus*	Ziconotide	Chronic pain	FDA (2004)
Urochordata	*Ecteinascidia turbinata*	Trabectedin, ET‐743	For advanced soft tissue tumors	FDA (2015)
	*Aplidium albicans*	Plitidepsin	Multiple myeloma and T‐cell lymphoma	TGA (2018)
	*Ecteinascidia turbinata*	Trabectedin	Soft tissue sarcoma, platinum‐sensitive recurrent ovarian cancer, and acute lymphoblastic leukemia	FDA (2015)
	*Ecteinascidia turbinata*	Lurbinectedin	Treatment of patients with small cell lung cancer progression after platinum‐based therapy	FDA (2020)
Fungus	*Streptomyces mediterranoi*	Rifampicin	Various tuberculosis	Italy (1968)
	*Cephalosporium acremonium*	Cephalosporin	Bacterial infection	FDA (1964)
Fish	Omega‐3‐acid ethyl esters	LOVAZA	Prevention of cardiovascular and cerebrovascular diseases and regulation of blood lipids	FDA (2004)
	Omega‐3‐fatty acids and EPA	VASCEPA	Hypertriglyceridemia	FDA (2013)
	Omega‐3‐fatty acids	EPANOVA	Hypertriglyceridemia	FDA (2014)
	Fish oil triglycerides	OMEGAVEN	Provide energy and fatty acids for children with parenteral nutrition‐related cholestasis	FDA (2018)
Arthropoda	*Limulus Polyphemus*	Keyhole limpet hemocyanin	Treatment of bladder cancer and solid tumor	Netherlands (1997)

**FIGURE 1:**
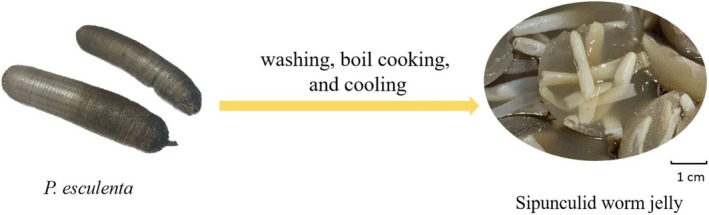
Physical pictures of *P. esculenta* and Sipunculid worm jelly.

*Phascolosoma esculenta* is an indigenous species of China, inhabiting the coastal regions of Fujian, Zhejiang, and Guangxi. Belonging to the phylum Sipuncula, class Phascolosomatidea, order Phascolosomaliformes, and family Phascolosomatidae, it thrives in the intertidal zone (Geng et al. 2016). With a cylindrical soft body typically measuring about 10 cm in length, it primarily feeds on seaweed and organic detritus. Renowned for its delectable flavor and nutritional richness, it is processed into a distinctive delicacy known as “Sipunculid worm jelly,” which is called as “Tu‐Sun‐Dong” in Chinese (Figure 1). It is known as the “ginseng of the sea” in Chinese folklore because of its effectiveness in enhancing endurance and immunity. In recent years, *P. esculenta* has gained significant attention as a delicious marine food with potential active value. This marine invertebrate is rich in protein and contains a variety of essential nutrients, including amino acids, fatty acids, and minerals. Compared to USDA data on whole eggs, it has higher levels of carbohydrates, protein, and minerals such as copper, iron, magnesium, phosphorus, potassium, selenium, sodium, and zinc (Wu, Fang, et al. 2014). Additionally, it contains a rich profile of amino acids, including eight essential amino acids and six flavor amino acids (Cai et al. 2020). The medicinal and culinary value of Sipuncula has been documented in the “Chinese Marine Materia Medica,” marking the first comprehensive compendium of marine pharmaceuticals in China. In recent years, numerous bioactive ingredients, including peptides (Du et al. 2013; Wu, Liu, et al. 2014; Wu, Fang, et al. 2014), polysaccharides (Wu et al. 2020), oligosaccharides (Yang et al. 2019), and enzyme (Cai, Zhou, et al. 2021; Liu et al. 2022; Niu et al. 2011; Wang, Su, et al. 2010) have been identified in *P. esculenta* . These ingredients have exhibited various pharmacological activities, including antibacterial effects (Liang et al. 2015; Shu et al. 2023; Zhang et al. 2020), hepatic and cardio‐cerebrovascular protection (Wu, Liu, et al. 2014; Wu, Fang, et al. 2014; Wu et al. 2020), antioxidant properties (Chen et al. 2021; Xing et al. 2024; Yang et al. 2019), antifatigue activity (Liu et al. 2016), and immune‐modulatory effects (Liang 2008). With the growing demand for health in human society, the potential value of *P. esculenta* as a health food is increasing. Therefore, many scholars have investigated the phylogeny, reproductive biology (Du et al. 2022; Gao et al. 2019; Hou et al. 2018; Long et al. 2015; Ying et al. 2009), and adaptability (Gao et al. 2012; Gu, Wang, et al. 2024; Hu et al. 2024; Ming, Wu, et al. 2021; You et al. 2019; Zheng et al. 2017) of *P. esculenta* to develop scientific strategies for scaling up the production of *P. esculenta* . This paper focuses on a review of the latest research status of the bioactive ingredients and physiological functions of *P. esculenta* , with the aim of highlighting its potential to be developed as functional health foods or even new drugs.

## 2 Bioactive Ingredients of *P. esculenta*

### 2.1 Ferritin

Ferritin is a kind of functional protein widely found in bacteria, plants, and animals, which plays an important role in iron metabolism, both in detoxifying intracellular iron and in storing iron (Arosio et al. 2009). Over 50 ferritin crystal structures have been identified, mostly from bacterial ferritins, and few of the nonbacterial ferritins are from marine invertebrates. For example, *Chaetopterus* ferritin from *Chaetopterus* sp. (parchment tube worm) was the first crystal of ferritin isolated from the marine invertebrates, but the mechanism by which it stores iron is unknown (De Meulenaere et al. 2017). Currently, two species of *P. esculenta* ferritin have been reported.

Du et al. acquired the gene sequence of *P. esculenta* ferritin (PeFer) using the RACE method. The sequence is 1017 bp long, comprising a 5′‐terminal noncoding region of 151 bp, a 3′‐noncoding region of 341 bp, and an open reading frame of 525 bp (including a stop codon), capable of encoding 175 amino acids, and the polyclonal antibody was prepared (Du et al. 2008; Su et al. 2009). Ding et al. conducted recombinant purification of PeFer in *Escherichia coli* and observed that under various heavy metal treatments, the recombinant PeFer exhibited distinct conformational changes and formed diverse aggregates, suggesting that PeFer may sequester different heavy metals through unique amino acid binding sites. Additionally, PeFer exhibits potent oxidative catalytic activity towards Fe and enrichment of toxic metal ions (Mn^2+^, Pb^2+^, Cd^2+^, Cr^3+^ and Cu^2+^, etc.) compared to nontoxic metal ions (Sn^2+^ and Zn^2+^) (Ding et al. 2018). Fer147, a new protein screened for interaction with PeFer using a yeast two‐hybrid system, has a full length of 916 bp, without a signal peptide, and it also belongs to the ferritin superfamily. The invertebrate ferritin contains only one subunit, whereas Fer147 has both the iron oxidase center of the H subunit and the iron nucleation site of the L subunit, indicating that Fer147 is functionally equivalent to both subunits of vertebrate ferritin (Ding et al. 2017). PeFer and Fer147(PDB: 6LPD, 6LPE) have similar structures to other known ferritins, but in terms of detailed structure, the triple channel of Fer147 and the quadruple channel of PeFer have different variations in electrostatic potential, and the two differ significantly in aggregating metal ions and excluding cations (Ming, Huan, et al. 2021). The enrichment ability of these two ferritins to heavy metals was better than that of natural horse‐spleen ferritin, and the enrichment ability of Fer147 to toxic heavy metals such as Cu^2+^, Pb^2+^ and Hg^2+^ was higher than that of PeFer (Ding et al. 2017). Through proteomic and metabolomic analyses, Ming et al. discovered that PeFer exerts a protective effect against Cd‐induced injury to bone marrow mesenchymal stem cells (BMSCs). It impedes BMSC proliferation by inducing G0/G1 cell cycle arrest and apoptosis, while safeguarding BMSCs from Cd‐induced apoptosis via energy metabolism (Ming, Wu, et al. 2021).

Iron ferritin is well known for its significant flexibility and adaptability in metal absorption, and it is considered a promising candidate for heavy metal detoxification and environmental detection agents. Furthermore, ferritin possesses a unique structure. A typical ferritin consists of 24 interconnected subunits arranged in the form of four‐helix bundles, creating a symmetrical hollow cage‐like shell with 4–3–2 symmetry that closely resembles a spherical shape. This shell exhibits excellent stability and is resistant to high temperatures, acids, and alkalis (Zang et al. 2017). Through a reduction reaction, the iron core in ferritin can be removed, resulting in the formation of apoferritin shells. Currently, nanotechnology based on ferritin has been developed, allowing the loading of small molecules into this shell‐like biological model to serve as carriers for novel bioinorganic nanoparticles (Li et al. 2007). As a naturally occurring nanocarrier in the environment, PeFer and Fer147 effectively reduce the quantity of organic solvents used in the preparation process, making it a potential environmentally friendly nanotechnology (Khoshnejad et al. 2018). Therefore, *P. esculenta* ferritin is expected to be developed as a natural nanoparticle carrier, with its strong ability to enrich metal ions and also making it suitable for transporting calcium, iron, and other essential elements required by the human body.

### 2.2 Trx‐Like Protein

Thioredoxin (Trx), a highly conserved antioxidant molecule, constitutes one of the principal antioxidant systems crucial for maintaining intracellular redox homeostasis. Excessive intracellular reactive oxygen species (ROS) can induce cellular oxidative damage or apoptosis. Cd^2+^, either directly or indirectly, can elevate intracellular ROS levels (Lu and Holmgren 2014). To elucidate the molecular response of Trx to Cd stress, Meng et al. identified a Trx isoform, named PeTrxl, from *P. esculenta* , aiming to enhance understanding of the role of Trx‐like protein 1 in intracellular ROS regulation under Cd stress. Experimental findings revealed the highest Trx levels in the body fluids of *P. esculenta* . The expression of PeTrxl mRNA markedly increased after 12 and 24 h of exposure to both low and high Cd concentrations, while the expression of Trx‐like protein 1 mRNA significantly decreased after 96 h of exposure. This suggests that Trx‐like protein 1 mRNA is responsive to Cd stress at early stages. The concentration of ROS in the coelomic fluid exhibited a notable increase subsequent to the administration of PeTrxl into *P. esculenta* . Insulin disulfide reduction and 2′2‐azino‐bis(3‐ethylbenzothiazoline‐6‐sulfonic acid) radical scavenging experiments suggested that PeTrxl may exert its antioxidant function by reducing disulfide bonds in proteins or directly scavenging ROS (Meng et al. 2021). Gu et al. studied the PeTrx2 gene from the mitochondria of *P. esculenta* . Under cadmium stress, PeTrx2 in the gut of *P. esculenta* exhibited a rapid response to oxidative stress. In vitro, recombinant rPeTrx2 demonstrated dose‐dependent REDOX activity, ABTS free radical scavenging ability, and enhanced cadmium tolerance in *E. coli* . After RNA interference with PeTrx2, proapoptotic genes (Caspase‐3 and Bax) were significantly upregulated, while antiapoptotic genes (Bcl‐2 and Bcl‐XL) were downregulated, confirming that PeTrx2 mitigates cadmium toxicity by regulating mitochondria‐dependent apoptotic pathways (Gu, Zheng, et al. 2024). Trx‐like protein 1 could serve as a biomarker for investigating the detoxification mechanism of invertebrate mudflat organisms exposed to heavy metals.

### 2.3 Hemerythrin

Hemerythrin serves as an oxygen carrier and was initially identified in marine invertebrates. In contrast to hemoglobin, hemerythrin utilizes a nonheme diiron active site, mitigating many stress‐induced side reactions associated with hemoglobin, including autoxidation and nitric oxide reaction (Fischer‐Fodor et al. 2011). Wang et al. generated a cDNA library of *P. esculenta* with a capacity of 3.06 × 105 cfu. The full‐length cDNA sequence of hemerythrin comprised 823 bp, encoding 120 amino acids. The protein had a molecular weight of 13.63 kDa and an isoelectric point of 5.78 (Wang, Li, et al. 2010; Wang, Su, et al. 2010). The sequencing of hemerythrin in *P. esculenta* serves as a valuable addition to hemerythrin research, facilitating further investigation into its potential as a raw material for artificial oxygen carriers, such as “blood substitutes.”

### 2.4 ACE Inhibitory Peptides

Hypertension is a prevalent clinical condition with an escalating incidence over time. The renin–angiotensin system, particularly the angiotensin I‐converting enzyme (ACE), plays a pivotal role in blood pressure regulation (Daskaya‐Dikmen et al. 2017). Synthetic ACE inhibitors have been developed, such as benazepril, enalapril, and perindopril, which have been shown to have good antihypertensive effects (Piepho 2000). However, these synthetic substances are prone to cause adverse side effects. There has been a trend to develop antihypertensive peptides from natural foods.

Du et al. isolated an ACE inhibitory peptide from *P. esculenta* with a molecular weight of 1222.7 Da and an amino acid sequence Ala‐Trp‐Leu‐His‐Pro‐Gly‐Ala‐Pro‐Lys‐Val‐Phe, suggesting that its ACE inhibitory activity may stem from the Phe residue at the C‐terminal and Ala residue at the N‐terminal, flanked by two positively charged amino acids, His and Lys (Du et al. 2013).

Wu et al. utilized BIOPEP to screen 22 proteins of *P. esculenta* and employed the LibDock module in Discovery Studio 3.5 software for molecular docking experiments to assess the inhibitory potential of the screened peptides on ACE. Ninety‐nine ACE inhibitory peptides exhibited IC_50_ values below 50 μM, and nine peptides were synthesized for validation. The IC_50_ values ranged from 3.43 to 4.38 μM, with an error margin of less than 1.0 unit (Wu, Liu, et al. 2014; Wu, Fang, et al. 2014).

Guo et al. isolated three ACE inhibitory peptides, RYDF (IC_50_ = 235 μM), YASGR (IC_50_ = 184 μM), and GNGSGYVSR (IC_50_ = 29 μM), with molecular weights of 600, 553, and 896, respectively, through pepsin–trypsin hydrolysis of water‐soluble proteins from *P. esculenta* . Automated molecular docking was employed to investigate the interaction between ACE and these peptides. GNGSGYVSR exhibited the highest ACE inhibitory activity and the lowest Ki value. It interacted with 7 hydrophobic residues and 12 hydrophilic residues of ACE, including Glu384, a critical residue for ACE‐Zn^2+^ binding. This peptide may enhance ACE inhibition by disrupting ACE‐Zn^2+^ binding. Compared to the other two peptides, GNGSGYVSR exhibited stronger and more stable residue binding, significantly reducing systolic blood pressure in spontaneously hypertensive rats from 228 mmHg to 197 mmHg at 2 h (*p* < 0.05) and maintaining this effect for 4 h. Thus, GNGSGYVSR emerges as a promising candidate for blood pressure regulation (Guo et al. 2017).

### 2.5 Fibrinolytic Enzyme

Fibrinolytic enzymes dissolve fibrin clots and are commonly employed as drugs to prevent thrombosis. Their advantage over anticoagulants and antiplatelet agents lies in their ability to directly target existing thrombi (Labrou 2019).

Current research on *P. esculenta* fibrinolytic enzyme focuses primarily on extraction and separation processes. Cai et al. employed homogenization, extraction, centrifugation, Sephadex G‐25 desalting, and DEAE Sepharose Fast Flow gradient elution to isolate *P. esculenta* fibrinolytic enzyme, which has a relative molecular mass of 32 Ku. *P. esculenta* fibrinolytic enzyme primarily resides in the intestinal tract. It exhibits some thermal stability but is sensitive to high temperatures, displaying optimal stability at 37°C and pH 6–9. Compared to the positive control urokinase, *P. esculenta* fibrinolytic enzyme exhibits direct fibrin degradation and thrombolytic effects (Cai, Zhou, et al. 2021; Cai, Xing, et al. 2021). *P. esculenta* fibrinolytic enzyme holds promise for the development of novel thrombolytic drugs.

### 2.6 Superoxide Dismutase

Superoxide dismutase (SOD) plays a crucial role in neutralizing superoxide anion radicals generated by extracellular stimuli and by‐products of oxygen metabolism from certain mitochondrial substrates (McCord and Fridovich 1969). Three isoforms of SOD have been identified: copper–zinc SOD (Cu/ZnSOD), manganese SOD (MnSOD), and extracellular SOD (Parge et al. 1992). *P. esculenta* possesses 12 copies of the Cu/ZnSOD gene and 15 copies of the MnSOD gene, the highest number of copies reported in the published annelid genome (Zhong et al. 2022).

Wang et al. isolated a complete MnSOD cDNA from *P. esculenta* . The cDNA spans 1385 bp, comprising an open reading frame of 681 bp that encodes 226 amino acids. The predicted protein has a molecular weight of 25.2 kDa and a theoretical isoelectric point of 5.96 (Wang, Li, et al. 2010; Wang, Su, et al. 2010). The Cu/ZnSOD cDNA, cloned by the Liu group, spans 857 bp, comprising a 75 bp 5' UTR, a 323 bp 3' UTR, and a 459 bp open reading frame, encoding 152 amino acids. The predicted molecular weight of the protein is approximately 15.6 kDa, with a theoretical isoelectric point of 5.65. The recombinant MnSOD from *P. esculenta* , expressed in *E. coli* , exhibited enhanced expression following exposure to heavy metal and temperature stress. Recombinant Cu/ZnSOD increases metal tolerance of *E. coli* under Cd stress, and shows antioxidant activity and scavenging ability of free radicals (Liu et al. 2022). Therefore, *P. esculenta* SODs can be used as indicators for the evaluation of heavy metals or developed as antioxidant drugs.

### 2.7 Polysaccharides

Aquatic organisms typically contain higher levels of polysaccharides compared to terrestrial counterparts. These polysaccharides exhibit anti‐inflammatory (Miller et al. 1993), anticoagulant (Mourão et al. 1996), antitumor (Hsu et al. 2018), and antioxidant effects (Li et al. 2015), rendering them valuable for development (Xiong et al. 2020).

Liang et al. determined that the optimal conditions for extracting *P. esculenta* polysaccharides were at 40°C extraction temperature, a phosphate buffer to raw material ratio of 2:1, an extraction time of 5.5 h, and a trypsin to raw material ratio of 1.6:1, resulting in the highest yield (Liang 2008). Wu et al. prepared *P. esculenta* polysaccharides by enzymatic digestion. The resulting *P. esculenta* polysaccharides exhibited a monosaccharide composition comprising mannose, ribose, rhamnose, glucuronide, glucose, galactose, xylose, arabinose, and caramel, with mass ratios approximately 3:2:1:1.6:7.6:5.5:1.5:1:3 (Wu et al. 2020). Additionally, *P. esculenta* polysaccharides exhibit notable in vitro free radical scavenging activity and can substantially impact lipid metabolism‐related indicators in hyperlipidemic mice, resulting in a significant hypolipidemic effect. PEP‐1 exhibited higher antioxidant capacity compared to PEP‐2, and it also enhanced the oxidative stress tolerance ability of *P. esculenta* . Furthermore, experiments revealed that mice with high‐fat‐induced liver conditions exhibited substantial fatty changes and cystic degeneration. However, these histopathological alterations were notably mitigated following treatment with *P. esculenta* polysaccharides, indicating a potential improvement in histological alterations. Zhou et al. isolated two polysaccharides, PEP‐1 and PEP‐2, from *P. esculenta* using column chromatography. These polysaccharides have molecular weights of 33.6 kDa and 5.7 × 10^3^ kDa, respectively. Their antioxidant capacities were evaluated in *Caenorhabditis elegans* , revealing that PEP‐1 exhibits superior efficacy compared to PEP‐2. Furthermore, PEP‐1 significantly enhances the oxidative stress tolerance of *C. elegans* (Zhou et al. 2024).

### 2.8 Oligosaccharides

Oligosaccharides, formed by the dehydration and condensation of 2–10 monosaccharide molecules or through the degradation of polysaccharides, exhibit high polarity and low degree of polymerization, often accompanied by specific pharmacological activities. Oligosaccharides have the characteristics of high polarity and low degree of polymerization, and often have certain pharmacological activities. Human milk oligosaccharides, for instance, promote infant intestinal and immune development (Donovan and Comstock 2016).

Yang et al. conducted the first preparation of *P. esculenta* oligosaccharides via enzymatic digestion. They characterized these oligosaccharides using liquid chromatography‐mass spectrometry and Fourier‐transform infrared, and established a mouse model of *E. coli* ‐induced sepsis to examine the protective effects of *P. esculenta* oligosaccharides. Analysis revealed that *P. esculenta* oligosaccharides primarily consist of D‐glucosyl and D‐galactosyl, with smaller amounts of D‐mannosyl and D‐arabinosyl. Sharp absorption peaks at 841.5 cm^−1^ and 892.5 cm^−1^ in the Fourier‐transform infrared spectra suggest the presence of both α‐type and β‐type glycosidic linkages in *P. esculenta* oligosaccharides. Mice with *E. coli* ‐induced sepsis, treated with *P. esculenta* oligosaccharides at all three doses, exhibited significantly increased survival rates and notable suppression of bacterial burden in the blood and liver. These results suggest that *P. esculenta* oligosaccharides may be enhancing survival through systemic bacterial clearance. Furthermore, administration of all three doses of *P. esculenta* oligosaccharides led to elevated levels of the anti‐inflammatory cytokine interleukin‐10 (IL‐10), accompanied by significant reductions in the secretion of pro‐inflammatory factors tumor necrosis factor‐alpha (TNF‐α) and interleukin‐1beta (IL‐1β) in mice receiving doses of 10 and 50 mg/kg. These findings indicate the potential of *P. esculenta* oligosaccharides to mitigate systemic and organ inflammation induced by *E. coli* infection through upregulation of IL‐10 expression, thereby reducing TNF‐α and IL‐1β‐related damage (Yang et al. 2019).

### 2.9 Summary Section

As above, the reported primary bioactive constituents of *P. esculenta* comprise polysaccharides, oligosaccharides, peptides, and functional proteins, as illustrated in Table 2. It is important to note that the current research on the composition of *P. esculenta* is far from sufficient. The discovery of new drugs and drug candidates from marine organisms is very promising, and many drugs based on marine organisms have been developed (Table 1). In the future, we can excavate more active compounds, including small molecule compounds, from the *P. esculenta* .

**TABLE 2:** Active ingredients of *Phascolosoma esculenta* and the functional values.

Bioactive ingredients	Pharmacological action	Potential value	Action mechanism	Reference
Ferritin	Protective effects against cadmium‐induced injury in bone marrow mesenchymal stem cells	Serving as a drug for heavy metal detoxification and an environmental detection agent	Inhibition of BMSC proliferation by inducing G0/G1 cell cycle arrest and apoptosis, and protection of BMSC from apoptosis through energy metabolism	Ding et al. (2018) and Ming, Wu, et al. (2021)
Trx‐like protein	Improve antioxidant capacity and protect *P. esculenta* from Cd‐induced oxidative stress	To offer insights for further investigation into the role of Trxl in mitigating Cd‐induced stress in marine invertebrates	PeTrxl antioxidant function may be exerted through the reduction of disulfide bonds in proteins or via direct reaction with ROS. PeTrx 2 (Caspase‐3、Bax↑ Bcl‐2, Bcl‐XL↓)	Gu, Zheng, et al. (2024); Meng et al. (2021)
Hemerythrin	/	As a raw material for artificial oxygen carriers (blood substitutes’).	/	Wang, Li, et al. (2010) and Wang, Su, et al. (2010)
ACE inhibitory peptides	ACE inhibitory activity. GNGSGYVSR reduced systolic blood pressure by 31 mmHg after 3 h	Develop as antihypertensive drugs or health products	The peptide forms hydrogen bonds with ACE residues. The more hydrogen bonds are formed, the stronger the binding force is	Guo et al. (2017) and Wu, Liu, et al. (2014) and Wu, Fang, et al. (2014)
Fibrinolytic enzyme	The blood clots of mice could be obviously dissolved in vitro, and the dissolution rate was 88.09% in 3 h	Developed as a thrombolytic drug.	/	Cai, Zhou, et al. (2021) and Cai, Xing, et al. (2021)
SOD	The content of ROS in coelomic fluid of *P. esculenta* was significantly decreased after injection of Pe‐Cu/Zn SOD protein	Evaluation of heavy metal indicators and development of antioxidant drugs	It participates in the detoxification of Cd by chelating heavy metal ions and scavenging reactive oxygen radicals	Liu et al. (2022)
Polysaccharides	It has hypolipidemic effect on hyperlipidemia mice	Develop as lipid‐lowering drugs or healthcare products	↓TC, TG, LDL‐C; MDA; ALT, AST ↑HDL‐C; SOD, GSH‐Px	Dong et al. (2018) and Wu et al. (2020)
Oligosaccharides	Significantly inhibit blood and liver bacterial load and improve the survival rate of septic mice	Development as a drug for the treatment of sepsis	↑IL‐10 ↓TNF‐α, IL‐β	Yang et al. (2019)

## 3 Physiological Functions of *P. esculenta*

**FIGURE 2:**
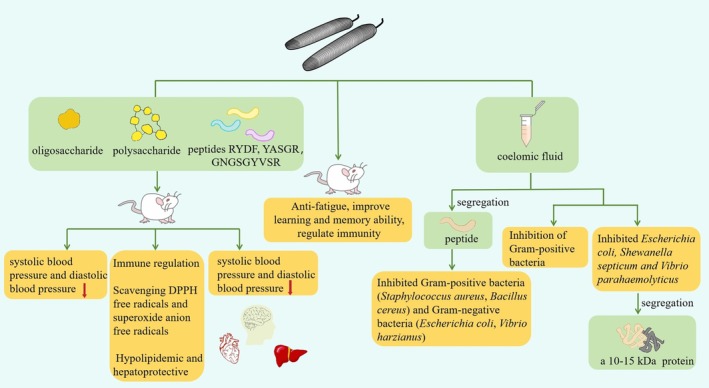
Various pharmacological activities of *P. esculenta*.

*Phascolosoma esculenta* serves not only as a nutritious food source but also holds medicinal properties such as nourishing “Qi and Yin” and strengthening the kidneys, as documented in ancient Chinese medical texts. It is utilized as a traditional Chinese medicine in select regions of China. The following summarizes the physiological functions of *P. esculenta* (Figure 2).

### 3.1 Antibacterial Activity

The coelomic fluid of *P. esculenta* harbors most of its antimicrobial active substances, whereas the body wall is typically consumed as food, leading to the underutilization of the coelomic fluid. Extracting antimicrobial agents from this fluid can optimize the utilization of *P. esculenta* , minimizing resource wastage and environmental pollution. Liang et al. employed three methods of direct centrifugation, ultrasonic centrifugation, and 2% acetic acid extraction to treat the body cavity fluid of *P. esculenta* , respectively. Among these, the supernatant obtained through ultrasonic centrifugation exhibited the most pronounced inhibitory effect on Gram‐positive bacteria, while its effects on Gram‐negative bacteria and fungi were less significant. The supernatant obtained by the other two methods showed no significant antibacterial activity (Liang et al. 2015). Meanwhile, Zhang et al. observed that *P. esculenta* coelomic fluid inhibited *E. coli* , *Shewanella septicum*, and *Vibrio parahaemolyticus* but had negligible effects on *Pseudomonas aeruginosa* , *Staphylococcus aureus* , *Bacillus subtilis* , and *Micrococcus lysodeikticus*. Isolation and purification from *P. esculenta* coelomic fluid yielded a 10–15 kDa small molecule active protein with antibacterial properties, likely constituting one of the antibacterial components in the organism (Zhang et al. 2020). Additionally, Shu et al. (Shu et al. 2023) isolated an antimicrobial peptide from the leukocytes of *P. esculenta* coelomic fluid, exhibiting efficacy against Gram‐positive bacteria ( *S. aureus* , *Bacillus cereus* ) and Gram‐negative bacteria ( *E. coli* , *Vibrio harveyi* ), along with robust resilience to high temperatures and alkalinity, and displaying nonhemolytic and protease resistance.

### 3.2 Cardio‐Cerebrovascular Protection

Wu et al. established a hyperlipidemia mouse model and observed that *P. esculenta* polysaccharides reduced total cholesterol, triglycerides, low‐density lipoprotein cholesterol, and the atherosclerosis index in the serum, indicating potential hypolipidemic effects. Moreover, *P. esculenta* polysaccharides increased serum SOD activity and reduced malondialdehyde (MDA) levels, thereby lowering serum free radicals and enhancing antioxidant capacity. Consequently, it is speculated that *P. esculenta* polysaccharides’ hypolipidemic function may be mediated through the antioxidant pathway (Wu et al. 2020).

### 3.3 Liver Protection

*Phascolosoma esculenta* polysaccharides significantly reduce liver coefficient, as well as levels of aspartate aminotransferase and alanine aminotransferase in liver tissue, promoting liver cell repair (Wu et al. 2020). Wu et al. conducted experiments using pepsin alone or in sequence with pepsin‐trypsin to digest water‐soluble and insoluble proteins from *P. esculenta* . They discovered that proteins digested with pepsin‐trypsin exhibited higher ACE inhibitory activity in vitro, with hydrolysates of insoluble proteins demonstrating greater ACE inhibitory activity than soluble ones. In vivo experiments on spontaneously hypertensive rats showed that pepsin‐trypsin hydrolysates significantly reduced both systolic and diastolic blood pressure, confirming their efficacy (Wu, Liu, et al. 2014; Wu, Fang, et al. 2014). Additionally, three peptides—RYDF, YASGR, and GNGSGYVSR—derived from *P. esculenta* also lowered systolic and diastolic blood pressure in spontaneously hypertensive rats, with GNGSGYVSR exhibiting the most pronounced effect, followed by YASGR, and RYDF showing a lesser effect (Guo et al. 2017). Cai et al. conducted in vitro experiments to assess the dissolution effect of fibrinolytic enzyme from *P. esculenta* on blood clots, revealing an 88.09% dissolution rate after 3 h (Cai, Zhou, et al. 2021; Cai, Xing, et al. 2021).

### 3.4 Antioxidant Activities

Chen et al. isolated proteins from five components of the luminal fluid of *P. esculenta* , focusing on the two proteins, PEP II and PEP V. The IC_50_ values of PEP II and PEP V for hydroxyl radicals were 1.637 and 0.998 mg/mL, respectively, while the IC_50_ values for 2,2‐diphenyl‐1‐picrylhydrazyl (DPPH) radicals were 5.581 and 2.801 mg/mL, respectively. The IC_50_ values for total reducing power were 3.700 and 1.461 mg/mL, respectively. Based on these three indices, both proteins exhibited significant antioxidant activity, though they showed considerable differences in DPPH radical clearance and total reducing power. Protein secondary structure analysis revealed that the secondary structure of PEP II consisted of β‐sheet (40.29%) > β‐turn (36.21%) > α‐helix (23.50%) > random (0%), while PEP V's secondary structure was composed of β‐turn (38.28%) > α‐helix (21.74%) > random (20.54%) > β‐sheet (19.44%). The authors further analyzed the relationship between secondary structure and antioxidant activity, speculating that β‐sheet may be associated with hydroxyl radical scavenging, while random coils and β‐turns appear to be positively correlated with DPPH radical clearance and total reducing power (Chen et al. 2021). Many compounds in *P. esculenta* display antioxidant capacities, such as *P. esculenta* polysaccharide, which exhibits significant scavenging activity for DPPH and superoxide anion radicals, potentially contributing to its hypolipidemic and hepatoprotective effects through antioxidant mechanisms (Wu et al. 2020). Oxidative stress is a crucial mechanism in sepsis pathogenesis. *P. esculenta* oligosaccharides enhanced glutathione peroxidase (GSH‐Px) and SOD enzyme activities while significantly inhibiting MDA activity in administered mice, demonstrating substantial antioxidant capacity and the ability to ameliorate oxidative stress. Both peptides and ferrous chelating‐peptides extracted from *P. esculenta* significantly enhanced the spatial learning memory of rats. Moreover, ferrous chelating‐peptides exhibited superior enhancement compared to peptides alone, indicating that chelation enhances peptide function. The impact of peptides and ferrous chelating‐peptides on spatial learning memory in mice may be attributed to peptides with a higher hydrophobic ratio affecting the in vivo antioxidant capacity of ferrous chelating‐peptides by stabilizing ROS (Yang et al. 2019).

### 3.5 Other Activities

*Phascolosoma esculenta* exhibits diverse activities including antifatigue effects, enhanced learning and memory, and immune regulation. Feeding mice with *P. esculenta* significantly increased liver glycogen and muscle glycogen content, resulting in prolonged swimming times (Niu and Tang 2012). Peptides and ferrous chelating‐peptides derived from *P. esculenta* significantly enhanced spatial learning and memory in rats, with ferrous chelating‐peptides showing superior efficacy to peptides, indicating the enhancing effect of chelation on peptides function. The improved spatial learning and memory associated with *P. esculenta* peptides and ferrous chelating‐peptides may be attributed to their high amino acid content and their ability to up‐regulate the expression of NR2A and NR2B, which are receptors related to learning and memory. Moreover, *P. esculenta* peptides can increase the expression of genes related to oxidative stress, thereby enhancing learning and memory. Additionally, *P. esculenta* peptides can enhance learning and memory by up‐regulating genes associated with oxidative stress (Liu et al. 2016). *P. esculenta* polysaccharides have been shown to increase the indices of liver, spleen, and thymus in mice, indicating its immune‐regulating function similar to many marine organisms (Liang 2008).

## 4 Adaptability of *P. esculenta*

*Phascolosoma esculenta* 's distinctive active ingredients and physiological functions hold potential for further development into dietary supplements or drugs. Nevertheless, the degradation of the ecological environment exposes the intertidal zones of estuaries and oceans to significant fluctuations, including easily fluctuating salinity levels (Du et al. 2021). These variations present a formidable challenge to the survival of invertebrates inhabiting the intertidal zones of oceans. The depletion of *P. esculenta* 's wild resources over time constrains its continued development and utilization. Consequently, researchers have investigated the resilience of *P. esculenta* to external factors such as heavy metals, temperature, and salinity, laying the groundwork for extensive artificial cultivation.

### 4.1 Adaptability to Heavy Metals

Heavy metal pollution is a prevalent environmental issue in marine ecosystems. Due to economic development and human activities, metals such as Cu, Pb, Ni, Cd, Zn, Fe, and Hg, released by industrial processes, have caused significant harm to both the environment and marine organisms (Traina et al. 2019). High concentrations of heavy metals can adversely affect *P. esculenta* . Gao et al. conducted an analysis of heavy metal content in sediment samples from various marine regions in Guangxi, Fujian, and Zhejiang. They discovered a higher distribution of essential trace elements such as Zn, Cu, Mn, and Fe in the muscle tissue of the species. Higher levels of Zn and Fe within the safe concentration range correspond to greater nutritional value. Additionally, traces of harmful elements such as Pb, As, Cd, and Hg were detected, albeit in small quantities. Among these metals, Cd and Hg exhibited the highest enrichment coefficients (Gao et al. 2012). Exposure to heavy metals Cd^2+^ and Hg^2+^ induced alterations in body fat, protein content, and fat content of body wall muscles. Higher metal concentrations resulted in more pronounced growth inhibition, and under the combined stress, the growth of *P. esculenta* almost stops. Nonetheless, in environments where metal stress exceeds 60 times the national fishery water quality standard, albeit with sluggish growth, mortality did not occur, suggesting robust metal tolerance in *P. esculenta* (Wu et al. 2015). This phenomenon may be associated with ferritin and metallothionein and antioxidant‐related mechanisms within the body of *P. esculenta* . Ferritin functions in heavy metal sequestration and detoxification, while metallothionein can provide protection against acute Cd poisoning. Notably, earthworms, also invertebrates, thrive in Cd‐rich soil, underscoring the crucial role of metallothionein. Nonetheless, studies on metallothionein in *P. esculenta* are lacking. Gu et al. identified a metallothionein (PeMT) and a metal‐responsive transcription factor 1 (PeMTF1) in *P. esculenta* . PeMT exhibited the highest expression in the gut, followed by the coelomic fluid, renal duct, constrictor muscle, and body wall. PeMTF1 was highly expressed in the body cavity fluid, contractile muscle, and intestinal tract. Under Zn stress, PeMT and PeMTF1 expression in the intestine of *P. esculenta* initially increased and then decreased. They then recombinantly expressed PeMT protein (PGX‐6P‐1‐MT), which significantly enhanced Zn tolerance in *E. coli* and exhibited dose‐dependent ABTS free radical scavenging activity. After RNA interference with PeMT, MDA content, SOD activity, GSH content, and Caspase‐3/8/9 activity significantly increased, indicating that PeMT plays a crucial role in chemical resistance and antiapoptotic processes. Furthermore, RNA interference with PeMTF1 led to a significant decrease in PeMT expression (by 53.95%), confirming the regulatory role of PeMTF1 in PeMT expression (Gu, Wang, et al. 2024). Furthermore, the accumulation of heavy metals by *P. esculenta* can serve as an indicator for ecological monitoring, facilitating the assessment of environmental metal pollution severity in coastal ecosystems.

### 4.2 Adaptability to Temperature

Ocean acidification and warming are poised to significantly impact the survival of planktonic larvae of marine invertebrates. However, marine species possess adaptive mechanisms to contend with environmental changes, including phenotypic plasticity or microgenetics. Particularly noteworthy is the adaptability of species inhabiting intertidal zones to fluctuations in temperature and pH (Foo and Byrne 2016). Nonetheless, extremes in temperature, whether too high or too low, can precipitate detrimental effects. Heat shock proteins (HSP) play a crucial role as molecular chaperones, being stress proteins synthesized by organisms in response to stress‐induced protein denaturation. Instances such as extreme cold temperatures, excessive cellular energy depletion, toxicity, and other extreme concentrations can trigger the expression of HSP. Invertebrates dwelling in the ocean's tidal zone undergo a substantial increase in body temperature following low tide, leading to the upregulation of HSP expression (Feder and Hofmann 1999). Among the various families of HSPs, HSP70 and HSP90 stand out; however, while HSP70 has been subject to extensive study in *P. esculenta* , research on HSP90 remains limited.

Li et al. conducted the cloning of HSP90 from *P. esculenta* , revealing a sequence spanning 2521 bp. This sequence encompasses a 5' untranslated region of 110 bp, a 3' untranslated region of 230 bp, and an open reading frame of 2181 bp. Investigation into the expression of *P. esculenta* HSP90 messenger RNA followed exposure to heavy metals and heat stress. The findings indicate that HSP90, belonging to the HSP90 family, exhibits high conservation levels and functions as an intracellular, non‐secretory protein. The upregulation of HSP90 expression occurred to varying degrees in response to the presence of Cd^2+^, Zn^2+^, and Cu^2+^, and elevated temperatures, suggesting its involvement in regulating heavy metal and heat stress in *P. esculenta* (Li et al. 2012). Su et al. similarly cloned the gene encoding HSP70 from *P. esculenta* . The cDNA sequence comprises 2520 bp, with a 5'‐terminal untranslated region of 125 bp, a 3'‐terminal untranslated region of 421 bp containing the typical polyadenylate signal sequence AATAAA, a poly(A) tail, and an open reading frame of 1974 bp. The expression of HSP70 in *P. esculenta* was induced by the presence of Zn^2+^, Cd^2+^, and elevated temperatures (Su et al. 2010).

Gao et al. conducted a study on the physiological and tissue changes in *P. esculenta* under acute heat stress. Following exposure to heat stress, there was a significant increase in the concentration of MDA and the activities of SOD and GSH‐Px in the coelomic fluid. Moreover, the expression of HSP70 and HSP90 in both the coelomic fluid and intestine was upregulated. Exposure to 40°C for 96 h resulted in visible cracks in the muscle layer of the tightly bound body wall and the stretched muscle layer, accompanied by alterations in the nuclei of muscle cells (Gao et al. 2022). Additionally, another investigation reported damage to the body wall and kidney of *P. esculenta* following 96 h of stress at 5°C. This stress led to the separation and rupture of the stratum corneum of the body wall and disordered arrangement of the single columnar epithelium in the renal epithelium. Furthermore, enlargement of the lumen formed by the outer membrane of the bottled protrusion and breakage of the circular muscle were observed (Shen et al. 2021). The expression levels of both HSP70 and HSP90 in *P. esculenta* were significantly elevated under conditions of both high and low temperature stress.

### 4.3 Adaptability to Salinity

Many marine invertebrates have developed adaptations to thrive in fluctuating salinity environments. However, high salinity estuaries exhibit low species richness, and species with high salt tolerance cannot survive below 5 ppt (Henry et al. 2012). In response to salinity stress, *P. esculenta* undergoes physiological adjustments, regulating enzyme activities and immune functions to mitigate damage induced by osmotic pressure fluctuations. The optimal salinity range for *P. esculenta* growth is 10–35 ppt, with peak activity observed at 25 ppt. Activity nearly ceases in both high and low salinity conditions, with the rhynchodaeum retracting (Zheng et al. 2017). Under low salt conditions, the activities of Na^+^/K^+^‐ATPase, acid phosphatase, and alkaline phosphatase initially increase and then decline before stabilizing (You et al. 2019). Low salt stress can induce renal muscle disorganization, necrosis, and expansion of the cytoplasmic sacs. When salinity is below 10‰, *P. esculenta* cannot survive. The lowest total SOD activity and highest MDA concentration were observed in the death group, indicating a breakdown of the antioxidant system and enhanced lipid peroxidation due to low salt stress. Transcriptomic analysis of differentially expressed genes suggested that low salt stress may lead to mortality through mechanisms such as disruption of ion transport and immune responses (Hu et al. 2024).

### 4.4 Adaptability to Hypoxic

Invertebrates inhabiting the intertidal zone of the ocean exhibit greater adaptability to anoxic‐reoxygenation conditions and demonstrate higher tolerance to hypoxic stress than other organisms (Ivanina et al. 2016). Xing et al. investigated the effects of hypoxic stress on the morphology, physiology, and biochemistry of *P. esculenta* . They found that after 7 days of hypoxia, the body turned black but did not die. Upon reoxygenation, the body became brown and malleable. Total antioxidant capacity (T‐AOC) increased, while MDA and lactate dehydrogenase (LDH) levels decreased, indicating that energy supply was maintained by enhancing antioxidant capacity and initiating anaerobic metabolism. Transcriptomic analysis of differentially expressed genes involved in immune response, carbon metabolism, apoptosis, and ribosomal function suggested that these may be key factors in the long‐term adaptation to low‐oxygen environments (Xing et al. 2024).

## 5 Strategies for the Research and Development of *P. esculenta*

**FIGURE 3:**
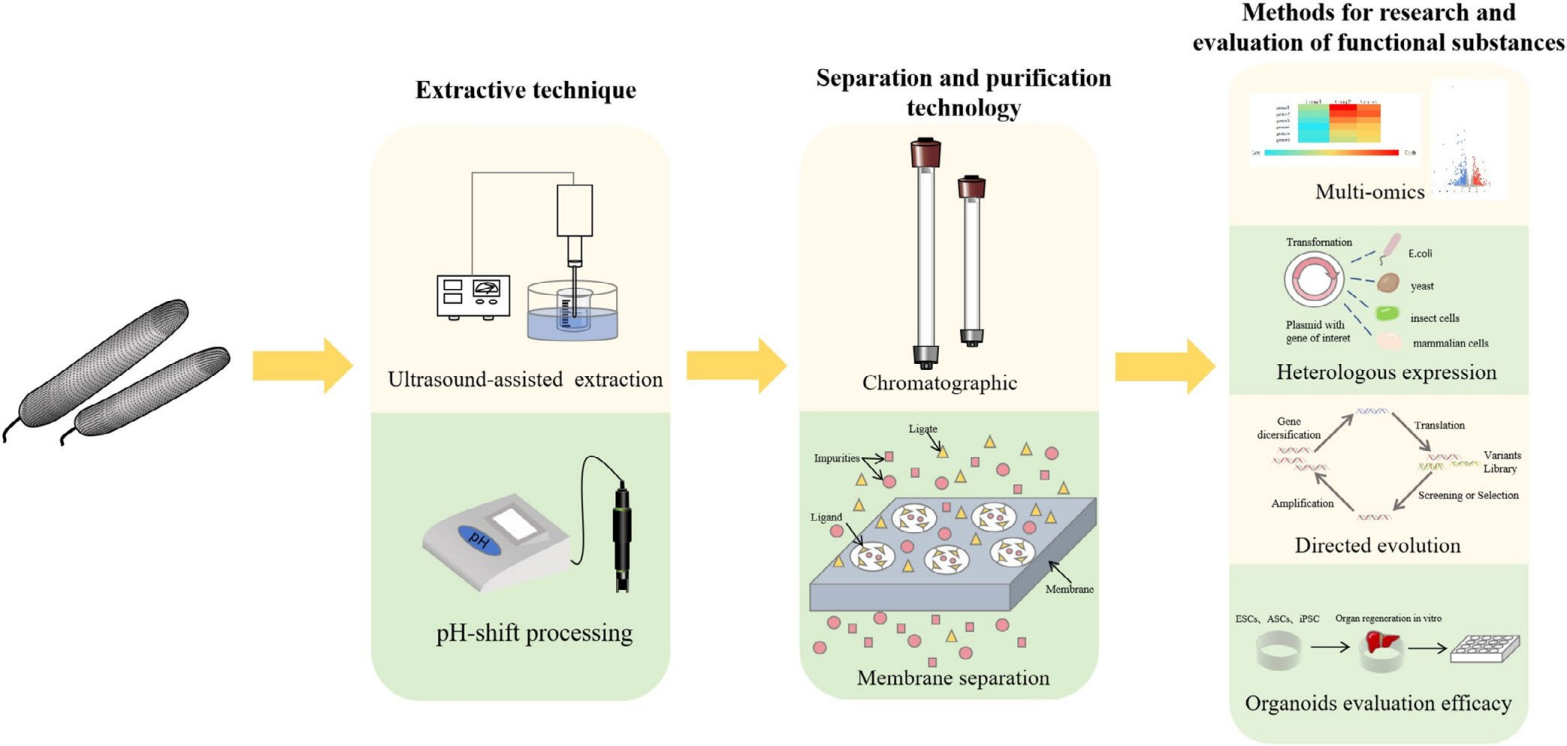
Existing or potential strategies and techniques for development of *P. esculenta*.

Despite its numerous potential benefits, research on *P. esculenta* remains notably inadequate. Given the growing demand for health products, the exploration of functional foods or novel drugs derived from *P. esculenta* could yield substantial economic rewards in the future. Sea cucumber, another marine food, is a well‐known medicinal and nutritional resource that has led to the development of numerous functional products and the establishment of a mature industrial chain (Hossain et al. 2020). Drawing upon the composition and activity traits of *P. esculenta* , this paper provides an overview of several technologies applicable to its research and development for future reference (Figure 3).

### 5.1 Extractive Techniques

#### 5.1.1 Ultrasound‐Assisted Extraction Technology

Ultrasound‐assisted extraction is a method employed to enhance extraction efficiency by utilizing ultrasound to disrupt biofilms such as cell membranes and cell walls. It is frequently combined with various techniques to improve the extraction rates of proteins and polysaccharides (Lin et al. 2023). Choi et al. extracted edible proteins from three insects using ultrasonic‐assisted extraction following degreasing with n‐hexane, resulting in a significant enhancement in protein yield (Choi et al. 2017). Görgüç, Özer et al. employed vacuum ultrasound‐assisted extraction and vacuum ultrasound‐assisted enzymatic extraction to extract proteins from sesame bran. Particularly noteworthy is the observation that under low vacuum conditions and short extraction times, combined vacuum ultrasound–assisted enzymatic extraction yielded the highest protein content and antioxidant capacity (Görgüç et al. 2020). Cheng et al. optimized the extraction conditions of *Moringa oleifera* leaf protein using ultrasonic‐microwave‐assisted extraction, resulting in improved extraction efficiency (Cheng et al. 2021). Beyond protein extraction, ultrasound‐assisted extraction technology can also be utilized for polysaccharide extraction. Qu et al. utilized a natural deep eutectic solvent for ultrasonic‐assisted extraction of abalone viscera polysaccharides. Comparisons with conventional methods revealed that the extracted polysaccharides exhibited higher sugar content, lower molecular weight, increased glucuronic acid content, and enhanced antioxidant capacity (Qu et al. 2023).

#### 5.1.2 pH‐Shift Processing

The pH‐shift process involves reducing the pH value to the vicinity of the protein's isoelectric point after mixing it within the optimal pH range of solubility, thereby rendering the protein insoluble and precipitated (Cavonius et al. 2015). Due to its simplicity, mild conditions, and ability to yield high‐quality protein, this method is commonly employed for extracting proteins with functional properties from by‐products such as fish, shrimp, and meat, facilitating the development of functional foods (Matak et al. 2015). For instance, valuable proteins can be extracted from low‐value herring light muscle and by‐product squid pen, thereby enhancing product utilization (Shavandi et al. 2017; Undeland et al. 2002).

### 5.2 Separation and Purification Technology

#### 5.2.1 Chromatographic Technique

Chromatography stands as one of the primary methods for investigating the material basis of natural products, commonly employed for the separation and purification of macromolecular substances. Contemporary chromatography methods encompass gel chromatography, affinity chromatography, ion exchange chromatography, and hydrophobic chromatography. Modern research frequently utilizes a combination of chromatography techniques to enhance product purity. For instance, Liu et al. isolated three bioactive peptides from *Cornu Bubali* (water buffalo horn) through a series of chromatographic steps, including gel filtration chromatography (Sephadex G‐25), ion exchange chromatography (DEAE Sepharose), and reverse phase chromatography (C18) (Liu et al. 2010). Similarly, Cao et al. isolated an active polypeptide from *Buthus martensi* Karch using gel column chromatography (Sephadex G‐50), cation exchange chromatography (CM‐Sephadex C‐50), and reversed phase chromatography (C18) (Cao et al. 2004).

#### 5.2.2 Membrane Separation Technique

Membrane separation technology is an efficient separation method characterized by high separation efficiency, low energy consumption, and environmental friendliness. It finds extensive applications in the biological and chemical industries. Based on the membrane mechanism, this technology is categorized into microfiltration, ultrafiltration, nanofiltration, and more. Currently, membrane separation technology is increasingly prevalent in biotechnology, particularly for the separation and purification of oligosaccharides, polysaccharides, and proteins (Saxena et al. 2009; Wang and Yu 2021). For instance, Jian et al. employed ultrafiltration technology to isolate konjac oligosaccharides with a molecular weight below 1000 Da from enzymatically degraded konjac (Jian et al. 2013). In another study, Cai et al. utilized multistage membrane separation technology to fractionate *Ganoderma lucidum* polysaccharides, obtaining polysaccharides with distinct molecular weights. They evaluated the activity of the isolated polysaccharides, comparing and screening those with the highest activity (Cai, Zhou, et al. 2021; Cai, Xing, et al. 2021). Membrane separation technology is integral in protein separation and purification processes, enabling the separation, concentration, and purification of proteins to enhance purity. With ongoing technological advancements, new filter membranes are emerging, holding the promise of becoming crucial tools in the industrial production of oligosaccharides, polysaccharides, and proteins in the future.

### 5.3 Methods for Mining and Evaluation of Bioactive Ingredients

#### 5.3.1 Multi‐Omics Techniques

In recent years, significant strides have been made in the genome sequencing of *P. esculenta* , culminating in the complete sequencing of its mitochondrial genome. However, there remains a need for further improvement in sequencing quality. Molecular phylogeny studies based on the mitochondrial genome post‐sequencing are scarce, as are investigations into the functional genome. Genome sequencing holds the potential to elucidate gene function, biochemistry, cellular processes, molecular mechanisms, and developmental pathways of *P. esculenta* , thus enhancing gene library quality. Through bioinformatics analysis, it can predict potential functional genomes and delve deeper into the molecular mechanisms underlying biological activities, thereby facilitating advancements in material basis research and quality evaluation (Zhang et al. 2022). Transcriptomics, which examines all RNA molecules within cells, serves to elucidate the functional elements of the genome and unveil the molecular composition of cells and tissues (Li et al. 2020). Transcriptomic studies of *P. esculenta* can be conducted through the following methods: mRNA extraction from crushed *P. esculenta* tissues, reverse transcription to synthesize cDNA using mRNA as a template, preparation of a cDNA library via PCR amplification, RNA‐seq sequencing, assembly of data into transcripts using assembly software, extraction of sequence information of active substances through translation, construction of the library, and verification of biological activity. For instance, Meng et al. utilized transcriptomic analysis to identify 13 cell populations in molluscan oysters *Crassostrea hongkongensis*, evaluating their specificity and identifying an immune‐activated granulocyte subgroup, hem_G0 (Meng et al. 2022).

Proteomics and peptidomics, based on extracted proteins and peptides, involve initial separation and purification, followed by enzymatic cleavage with specific proteases. Subsequently, they are analyzed using high‐resolution mass spectrometry to obtain secondary fragment information. The acquired data are then compared with protein polypeptide databases to identify the components of proteins and peptides. Finally, establishing a biological evaluation model clarifies the pharmacodynamic material basis of the purified or synthesized protein polypeptide monomer. For instance, proteomics was employed to investigate the venom components of *Deinagkistrodon acutus* , resulting in the identification of 29 different proteins belonging to eight families of snake venom proteins. This contributes to a better understanding of the pathophysiology of poisoning caused by *D. acutus* toxicity (Chen et al. 2019). Li et al. employed quantitative proteomics to investigate the effects of cold stress on the composition, structure, and physical properties of myofibrillar protein in *Procambarus clarkii* . They identified two proteins that may play a key role in the hardening process of shrimp meat under cold stress (Li et al. 2022).

Currently, single‐omics technology falls short in revealing biological functions comprehensively. However, through multi‐omics integration and correlation, a holistic approach is employed to observe and analyze, addressing key scientific inquiries. Leveraging advancements in high‐throughput sequencing, the utilization of high‐resolution mass spectrometry, and the continuous enrichment of databases, multi‐omics technology enables the analysis of organism function and mechanisms through a systemic biology perspective (Bennett et al. 2023; Koh et al. 2019). In the future, *P. esculenta* can leverage multi‐omics techniques to unearth additional active ingredients and elucidate their molecular mechanisms.

#### 5.3.2 Bioengineering Technology

The use of various omics techniques to identify target functional proteins and peptides, followed by cloning and heterologous expression through bioengineering methods, is a commonly employed approach for obtaining sufficient quantities for activity screening and functional studies (Rivera‐de‐Torre et al. 2022). The initial step involves selecting an appropriate expression system. Currently, the commonly utilized prokaryotic expression system is *E. coli* , known for its convenience, cost‐effectiveness, and efficiency, making it the primary choice for prokaryotic protein production. Eukaryotic expression systems encompass yeast, insect cells, or mammalian cells. Yeast expression facilitates essential posttranslational modifications, while insect cell expression stands as a prevalent heterologous system in both scientific research and industry, particularly for membrane protein expression (Adam et al. 2023; Ramya et al. 2011). Mammalian cells are preferred for producing larger or more intricate eukaryotic proteins (Hunter et al. 2019). Typically, *E. coli* strains are chosen for prokaryotic protein expression, while the selection of yeast, insect cells, or mammalian cells depends on the specific requirements for posttranslational modifications, especially concerning sugar chains, in eukaryotic proteins (Schutz et al. 2023).

#### 5.3.3 Protein Engineering Technique

Protein engineering technology enables the design of proteins at the molecular level and the mutation of proteins through DNA recombination to yield functional proteins aligned with desired specifications. Directed evolution, a method for gene‐editing proteins, has gradually emerged in recent decades. By mimicking the natural evolution process in laboratory settings, it facilitates the transformation of target biomolecules, thereby enabling the acquisition of more specific proteins post‐optimization and transformation. This approach holds great promise in drug research, as well as in the field of biological food and beyond (Wang et al. 2021). Semi‐rational design, which is based on directed evolution, can also be employed to construct a smaller and higher quality mutant library by selectively targeting and transforming biomolecules in a nonrandom manner (Lutz 2010). *P. esculenta* harbors a variety of active proteins that have been identified. Protein engineering technology is leveraged to modify and optimize these proteins, amplifying their advantages or uncovering novel functionalities.

#### 5.3.4 Evaluation Method of Functional Substances

Unlike traditional animal models, which cannot assess whether an effective substance undergoes changes after ingestion, primates are the most appropriate models for drug evaluation due to their close resemblance to humans, despite being time consuming and expensive (Mou et al. 2023). The evaluation of effective substances using cell models also faces challenges in directly reflecting their functional efficacy. Thus, there is a pressing need to develop a novel activity evaluation system that comprehensively assesses biological effects. Apart from conventional models like cells and animals, organoids offer distinct advantages such as high fidelity and stable propagation, with a composition and structure more akin to human organs. By simulating organ function in vivo, testing the mode of action of active ingredients in vitro can more accurately mirror the developmental trajectory of human organs. This emerging alternative technology holds promise for reducing reliance on animal experimentation (Schutgens and Clevers 2020). Consequently, organoids can serve as a screening platform for evaluating the activity of *P. esculenta* components. Particularly when the number of isolated peptides is insufficient, the organoid platform can facilitate preliminary screening, guiding the scalable production of active peptides and proteins using biotechnology.

### 5.4 Food Development Strategy

Currently, no studies have confirmed whether the activity of *P. esculenta* affects the quality of bioactive substances in the body. Nonetheless, *P. esculenta* is anticipated to evolve into a functional food with medicinal properties. Maintaining ingredient freshness is crucial for optimal taste. Concerns also arise regarding the preservation of active ingredients during transportation, storage, and production of marketable products. Various storage technologies are available for this purpose. Ultra‐high pressure technology, a non‐thermal food preservation method, utilizes liquid as a pressure transfer medium to deactivate food‐borne pathogens and enzymes in pressure‐treated food. This technology has seen widespread adoption in the food industry in recent years (Wang et al. 2016). For instance, treating frozen pink salmon fillets with ultra‐high pressure technology minimally affects their odor while reducing the resistance of pathogens like *Listeria monocytogenes* and *Salmonella enterica* (Boziaris et al. 2021). Méndez et al. observed that ultra‐high pressure treatment inhibits lipid hydrolysis in sardines without altering the activity of enzymes such as acid phosphatase and cathepsin B and D, and without causing significant changes in sarcoplasmic and myofibrillar proteins (Méndez et al. 2017). Given its pronounced seafood flavor, *P. esculenta* holds promise as a seafood seasoning. Even discarded parts like coelomic fluid can be repurposed. Utilizing the Maillard reaction to create condiments from *P. esculenta* and its by‐products can recover nutrients from these by‐products, enhance the value of *P. esculenta* , and introduce new products, thereby addressing issues of low product utilization to some extent (Wang et al. 2019).

## 6 Conclusions

*Phascolosoma esculenta* boasts a wealth of nutrients and encompasses diverse bioactive substances, including polysaccharides, peptides, enzymes, and various functional proteins. These bioactive components manifest antibacterial, hepatoprotective, cardio‐cerebrovascular protective, antioxidant, antifatigue, and immune‐regulatory activities. However, there is relatively little research on its bioactive components and functional properties, leaving substantial room for exploration. Furthermore, compared to many Sipuncula species, its chemical diversity remains largely unexplored. Six novel linear guanidine amides were recently isolated from *P. granulatum* , an asteroid species, utilizing mass spectrometry and nuclear magnetic resonance techniques (Jennings et al. 2024). This approach may also facilitate the exploration of additional compounds derived from *P. esculenta* .

The present focus on the development of animal and marine biological medicine is hindered by a severe shortage of resources. Ongoing studies on the phylogenetic development, reproduction, and tolerance of *P. esculenta* lay the groundwork for its artificial breeding. The species holds considerable potential across industries such as food, medicine, nutrition, healthcare, and environmental monitoring. Developing high‐value functional foods or adjuvant therapy drugs based on *P. esculenta* is promising. This paper consolidates information on the active ingredients, physiological functions, and adaptability of *P. esculenta* , providing a foundation for further exploration. In addition to the biological components such as protein and polysaccharide, future investigations could also emphasize the small molecular active substances of *P. esculenta* . Various biological techniques can be employed to unlock the species' maximum economic value.

## Author Contributions

**Lingxuan Chen:** writing – original draft (equal). **Wen Xu:** writing – review and editing (supporting). **Rui Zhu:** writing – review and editing (supporting). **Shaohua Xu:** writing – review and editing (equal). **Hua Li:** writing – review and editing (equal). **Lixia Chen:** writing – review and editing (equal).

## Conflicts of Interest

The authors declare no conflicts of interest.

## Data Availability

No data were used for the research described in the article.

## References

[fsn370217-bib-0089] Adam, T. , M. L. Dylan , and G. Angie . 2023. “Optimized Expression and Isolation of Recombinant Active Secreted Proteases Using Pichia Pastoris.” Bio‐Protocol 13, no. 5: e4628. 10.21769/BioProtoc.4628. 36908634 PMC9993078

[fsn370217-bib-0001] Arosio, P. , R. Ingrassia , and P. Cavadini . 2009. “Ferritins: A Family of Molecules for Iron Storage, Antioxidation and More.” Biochimica et Biophysica Acta, General Subjects 1790, no. 7: 589–599. 10.1016/j.bbagen.2008.09.004. 18929623

[fsn370217-bib-0002] Bennett, H. , W. Stephenson , C. Rose , and S. Darmanis . 2023. “Single‐Cell Proteomics Enabled by Next‐Generation Sequencing or Mass Spectrometry.” Nature Methods 20, no. 3: 363–374. 10.1038/s41592-023-01791-5. 36864196

[fsn370217-bib-0003] Boziaris, I. , F. Parlapani , and C. DeWitt . 2021. “High Pressure Processing at Ultra‐Low Temperatures: Inactivation of Foodborne Bacterial Pathogens and Quality Changes in Frozen Fish Fillets.” Innovative Food Science & Emerging Technologies 74: 102811. 10.1016/j.ifset.2021.102811.

[fsn370217-bib-0004] Buchanan, R. A. , and F. Hess . 1980. “Vidarabine (Vira‐A): Pharmacology and Clinical Experience.” Pharmacology & Therapeutics 8, no. 1: 143–171. 10.1016/0163-7258(80)90063-7.

[fsn370217-bib-0005] Cai, B. , F. Zhou , W. Huang , and S. Ruan . 2020. “Nutritional Evaluation and Volatile Flavor Compounds Analysis of Cultured and Wild *Phascolosoma esculenta* .” Science and Technology of Food Industry 41, no. 21: 240–245. 10.13386/j.issn1002-0306.2020020034.

[fsn370217-bib-0006] Cai, B. , F. Zhou , S. Ruan , W. Huang , C. Liu , and Y. Su . 2021. “Isolation and Characterization of a Fibrinolytic Enzyme From Cultured Sipunculid *Phasclosoma esculenta* .” Fisheries Science 40, no. 6: 922–928. 10.16378/j.cnki.1003-1111.20053.

[fsn370217-bib-0007] Cai, M. , H. Xing , B. Tian , et al. 2021. “Characteristics and Antifatigue Activity of Graded Polysaccharides From *Ganoderma lucidum* Separated by Cascade Membrane Technology.” Carbohydrate Polymers 269: 118329. 10.1016/j.carbpol.2021.118329. 34294340

[fsn370217-bib-0008] Cao, Z. Y. , Z. M. Mi , G. F. Cheng , et al. 2004. “Purification and Characterization of a New Peptide With Analgesic Effect From the Scorpion *Buthus martensi* Karch.” Journal of Peptide Research 64, no. 1: 33–41. 10.1111/j.1399-3011.2004.00164.x. 15200476

[fsn370217-bib-0009] Cavonius, L. , E. Albers , and I. Undeland . 2015. “pH‐Shift Processing of Nannochloropsis Oculata Microalgal Biomass to Obtain a Protein‐Enriched Food or Feed Ingredient.” Algal Research‐Biomass Biofuels and Bioproducts 11: 95–102. 10.1016/j.algal.2015.05.022.

[fsn370217-bib-0010] Chen, P. , M. Huang , J. Chang , C. Liu , C. Chen , and C. Hsieh . 2019. “Snake Venom Proteome and Immuno‐Profiling of the Hundred‐Pace Viper, *Deinagkistrodon acutus*, in Taiwan.” Acta Tropica 189: 137–144. 10.1016/j.actatropica.2018.09.017. 30268686

[fsn370217-bib-0011] Chen, Q. , H. Chi , J. Qiu , et al. 2021. “Extraction and Antioxidant Properties of Protein From *Phascolosoma esculenta* Coelomic Fluid.” Food Science and Technology 46, no. 7: 155–160. 10.13684/j.cnki.spkj.2021.07.026.

[fsn370217-bib-0012] Cheng, F. , G. Shu , L. Chen , et al. 2021. “Ultrasound‐Microwave Assisted Extraction of Proteins From *Moringa oleifera* Leaves: Comparative Optimization Study and LC‐MS Analysis of the Protein Concentrate.” Journal of Food Processing and Preservation 45, no. 6: e15547. 10.1111/jfpp.15547.

[fsn370217-bib-0013] Choi, B. , N. Wong , and J. Auh . 2017. “Defatting and Sonication Enhances Protein Extraction From Edible Insects.” Food Science of Animal Resources 37, no. 6: 955–961. 10.5851/kosfa.2017.37.6.955. PMC5932934 29725219

[fsn370217-bib-0014] Daskaya‐Dikmen, C. , A. Yucetepe , F. Karbancioglu‐Guler , H. Daskaya , and B. Ozcelik . 2017. “Angiotensin‐I‐Converting Enzyme (ACE)‐Inhibitory Peptides From Plants.” Nutrients 9, no. 4: 316. 10.3390/nu9040316. 28333109 PMC5409655

[fsn370217-bib-0015] De Meulenaere, E. , J. Bailey , F. Tezcan , and D. Deheyn . 2017. “First Biochemical and Crystallographic Characterization of a Fast‐Performing Ferritin From a Marine Invertebrate.” Biochemical Journal 474, no. 24: 4193–4206. 10.1042/BCJ20170681. 29127253

[fsn370217-bib-0016] Ding, H. , D. Zhang , S. Chu , J. Zhou , and X. Su . 2017. “Screening and Structural and Functional Investigation of a Novel Ferritin From *Phascolosoma esculenta* .” Protein Science 26, no. 10: 2039–2050. 10.1002/pro.3241. 28726294 PMC5606535

[fsn370217-bib-0017] Ding, H. , J. Zhou , Y. Han , and X. Su . 2018. “Characterization of Recombinant *Phascolosoma esculenta* Ferritin as an Efficient Heavy Metal Scavenger.” Protein and Peptide Letters 25, no. 8: 767–775. 10.2174/0929866525666180806111756. 30081783

[fsn370217-bib-0018] Dong, Y. , Y. Qi , M. Liu , et al. 2018. “Antioxidant, Anti‐Hyperlipidemia and Hepatic Protection of Enzyme‐Assisted *Morehella esculenta* Polysaccharide.” International Journal of Biological Macromolecules 120: 1490–1499. 10.1016/j.ijbiomac.2018.09.134. 30266646

[fsn370217-bib-0019] Donovan, S. M. , and S. S. Comstock . 2016. “Human Milk Oligosaccharides Influence Neonatal Mucosal and Systemic Immunity.” Annals of Nutrition & Metabolism 69: 42–51. 10.1159/000452818. 28103609 PMC6392703

[fsn370217-bib-0020] Du, C. , D. L. Mu , X. M. Gao , et al. 2022. “Expression Characteristics and Putative Functions of KIF3A/KIF3B During Spermiogenesis of *Phascolosoma esculenta* .” Journal of Ocean University of China 21, no. 4: 998–1016. 10.1007/s11802-022-4881-x.

[fsn370217-bib-0021] Du, J. , K. Park , C. Jensen , T. M. Dellapenna , W. G. Zhang , and Y. Shi . 2021. “Massive Oyster Kill in Galveston Bay Caused by Prolonged Low‐Salinity Exposure After Hurricane Harvey.” Science of the Total Environment 774: 145132. 10.1016/j.scitotenv.2021.145132.

[fsn370217-bib-0022] Du, L. , M. Fang , H. Wu , et al. 2013. “A Novel Angiotensin I‐Converting Enzyme Inhibitory Peptide From *Phascolosoma esculenta* Water‐Soluble Protein Hydrolysate.” Journal of Functional Foods 5, no. 1: 475–483. 10.1016/j.jff.2012.12.003.

[fsn370217-bib-0023] Du, L. L. , T. W. Li , X. R. Su , and D. F. Li . 2008. “Cloning and Sequencing of Ferritin Gene of *Phascooloma esculenta* .” Oceanologia et Limnologia Sinica 39, no. 3: 252–256. 10.3321/j.issn:0029-814X.2008.03.010.

[fsn370217-bib-0024] Feder, M. E. , and G. E. Hofmann . 1999. “Heat‐Shock Proteins, Molecular Chaperones, and the Stress Response: Evolutionary.” Annual Review of Physiology 61, no. 61: 243–282. 10.1146/annurev.physiol.61.1.243. 10099689

[fsn370217-bib-0025] Fischer‐Fodor, E. , A. Mot , F. Deac , M. Arkosi , and R. Silaghi‐Dumitrescu . 2011. “Towards Hemerythrin‐Based Blood Substitutes: Comparative Performance to Hemoglobin on Human Leukocytes and Umbilical Vein Endothelial Cells.” Journal of Biosciences 36, no. 2: 215–221. 10.1007/s12038-011-9066-5. 21654075

[fsn370217-bib-0026] Foo, S. A. , and M. Byrne . 2016. “Acclimatization and Adaptive Capacity of Marine Species in a Changing Ocean.” Advances in Marine Biology 74: 69–116. 10.1016/bs.amb.2016.06.001. 27573050

[fsn370217-bib-0027] Gao, X. , D. Mu , C. Hou , J. Zhu , S. Jin , and C. Wang . 2019. “Expression and Putative Functions of KIFC1 for Nuclear Reshaping and Midpiece Formation During Spermiogenesis of *Phascolosoma esculenta* .” Gene 683: 169–183. 10.1016/j.gene.2018.10.021. 30316921

[fsn370217-bib-0028] Gao, X. , H. Yang , D. Tang , et al. 2022. “Physiological and Histological Responses of *Phascolosoma esculenta* (Sipuncula: Phascolosomatidea) to Acute Heat Stress.” Journal of Oceanology and Limnology 40, no. 2: 643–655. 10.1007/s00343-021-1013-1.

[fsn370217-bib-0029] Gao, Y. , L. Pan , H. Wu , and Z. Wang . 2012. “Contents and Correlationship of Heavy Metals in *Phascolosoma esculentas* and Their Habitat Sediments.” Marine Sciences 36, no. 10: 54–60.

[fsn370217-bib-0030] Geng, X. , M. Boufadel , and N. Jackson . 2016. “Evidence of Salt Accumulation in Beach Intertidal Zone due to Evaporation.” Scientific Reports 6: 31486. 10.1038/srep31486. 27511713 PMC4980607

[fsn370217-bib-0031] Glantz, M. J. , S. LaFollette , K. A. Jaeckle , et al. 1999. “Randomized Trial of a Slow‐Release Versus a Standard Formulation of Cytarabine for the Intrathecal Treatment of Lymphomatous Meningitis.” Journal of Clinical Oncology 17, no. 10: 3110–3116. 10.1200/JCO.1999.17.10.3110. 10506606

[fsn370217-bib-0032] Görgüç, A. , P. Özer , and F. M. Yilmaz . 2020. “Simultaneous Effect of Vacuum and Ultrasound Assisted Enzymatic Extraction on the Recovery of Plant Protein and Bioactive Compounds From Sesame Bran.” Journal of Food Composition and Analysis 87: 103424. 10.1016/j.jfca.2020.103424.

[fsn370217-bib-0033] Gu, S. W. , J. Q. Wang , X. M. Gao , et al. 2024. “Expression and Functional Analysis of the Metallothionein and Metal‐Responsive Transcription Factor 1 in *Phascolosoma esculenta* Under Zn Stress.” International Journal of Molecular Sciences 25, no. 13: 7368. 10.3390/ijms25137368. 39000475 PMC11242308

[fsn370217-bib-0034] Gu, S. W. , X. B. Zheng , X. M. Gao , Y. Liu , Y. E. Chen , and J. Q. Zhu . 2024. “Cadmium‐Induced Oxidative Damage and the Expression and Function of Mitochondrial Thioredoxin in *Phascolosoma esculenta* .” International Journal of Molecular Sciences 25, no. 24: 13283. 10.3390/ijms252413283. 39769049 PMC11676412

[fsn370217-bib-0035] Guo, M. , X. Chen , Y. Wu , et al. 2017. “Angiotensin I‐Converting Enzyme Inhibitory Peptides From Sipuncula (*Phascolosoma esculenta*): Purification, Identification, Molecular Docking and Antihypertensive Effects on Spontaneously Hypertensive Rats.” Process Biochemistry 63: 84–95. 10.1016/j.procbio.2017.08.009.

[fsn370217-bib-0036] Haque, N. , S. Parveen , T. T. Tang , J. E. Wei , and Z. N. Huang . 2022. “Marine Natural Products in Clinical Use.” Marine Drugs 20, no. 8: 528. 10.3390/md20080528. 36005531 PMC9410185

[fsn370217-bib-0037] Henry, R. P. , Č. Lucu , H. Onken , and D. Weihrauch . 2012. “Multiple Functions of the Crustacean Gill: Osmotic/Ionic Regulation, Acid‐Base Balance, Ammonia Excretion, and Bioaccumulation of Toxic Metals.” Frontiers in Physiology 3: 431. 10.3389/fphys.2012.00431. 23162474 PMC3498741

[fsn370217-bib-0038] Hossain, A. , D. Dave , and F. Shahidi . 2020. “Northern Sea Cucumber (*Cucumaria frondosa*): A Potential Candidate for Functional Food, Nutraceutical, and Pharmaceutical Sector.” Marine Drugs 18, no. 5: 274. 10.3390/md18050274. 32455954 PMC7281287

[fsn370217-bib-0039] Hou, C. C. , X. M. Gao , J. Ni , et al. 2018. “The Expression Pattern and Potential Functions of PHB in the Spermiogenesis of *Phascolosoma esculenta* .” Gene 652: 25–38. 10.1016/j.gene.2018.01.056. 29360606

[fsn370217-bib-0040] Hsu, H. , T. Lin , C. Hu , D. Shu , and M. Lu . 2018. “Fucoidan Upregulates TLR4/CHOP‐Mediated Caspase‐3 and PARP Activation to Enhance Cisplatin‐Induced Cytotoxicity in Human Lung Cancer Cells.” Cancer Letters 432: 112–120. 10.1016/j.canlet.2018.05.006. 29746926

[fsn370217-bib-0041] Hu, P. F. , C. Y. Wang , T. Y. Zhao , et al. 2024. “Transcriptome, Antioxidant Enzymes and Histological Analysis Reveal Molecular Mechanisms Responsive to Low Salinity Stress in *Phascolosoma esculenta* .” Aquaculture Reports 34: 101884. 10.1016/j.aqrep.2023.101884.

[fsn370217-bib-0042] Hunter, M. , P. Yuan , D. Vavilala , and M. Fox . 2019. “Optimization of Protein Expression in Mammalian Cells.” Current Protocols in Protein Science 95, no. 1: e77. 10.1002/cpps.77. 30265450

[fsn370217-bib-0043] Ivanina, A. V. , I. Nesmelova , L. Leamy , E. P. Sokolov , and I. M. Sokolova . 2016. “Intermittent Hypoxia Leads to Functional Reorganization of Mitochondria and Affects Cellular Bioenergetics in Marine Molluscs.” Journal of Experimental Biology 219, no. 11: 1659–1674. 10.1242/jeb.134700. 27252455

[fsn370217-bib-0044] Jennings, L. K. , N. Kaur , M. C. Ramos , F. Reyes , M. M. Reddy , and O. P. Thomas . 2024. “Highly Concentrated Linear Guanidine Amides From the Marine Sipunculid *Phascolosoma granulatum* .” Journal of Natural Products 87, no. 4: 906–913. 10.1021/acs.jnatprod.3c01186. 38430199 PMC11061827

[fsn370217-bib-0045] Jian, W. , Y. Sun , H. Huang , et al. 2013. “Study on Preparation and Separation of Konjac Oligosaccharides.” Carbohydrate Polymers 92, no. 2: 1218–1224. 10.1016/j.carbpol.2012.09.065. 23399149

[fsn370217-bib-0046] Khoshnejad, M. , H. Parhiz , V. Shuvaev , I. Dmochowski , and V. R. Muzykantov . 2018. “Ferritin‐Based Drug Delivery Systems: Hybrid Nanocarriers for Vascular Immunotargeting.” Journal of Controlled Release 282: 13–24. 10.1016/j.jconrel.2018.02.042. 29522833 PMC6008199

[fsn370217-bib-0047] Koh, H. W. L. , D. Fermin , C. Vogel , K. P. Choi , R. M. Ewing , and H. Choi . 2019. “iOmicsPASS: Network‐Based Integration of Multiomics Data for Predictive Subnetwork Discovery.” Npj Systems Biology and Applications 5: Article 22. 10.1038/s41540-019-0099-y. PMC6616462 31312515

[fsn370217-bib-0048] Labrou, N. 2019. Therapeutic Enzymes: Function and Clinical Implications Preface (Vol. 1148). Advances in Experimental Medicine and Biology. Springer Singapore.

[fsn370217-bib-0049] Li, C. H. , X. R. Su , M. Q. Wang , et al. 2012. “Alteration of *Phascolosoma esculenta* Heat Shock Protein 90 Expression Under Heavy Metal Exposure and Thermal Stress.” Genetics and Molecular Research 11, no. 3: 2641–2651. 10.4238/2012.July.10.14. 22869079

[fsn370217-bib-0050] Li, M. , C. Viravaidya , and S. Mann . 2007. “Polymer‐Mediated Synthesis of Ferritin‐Encapsulated Inorganic Nanoparticles.” Small 3, no. 9: 1477–1481. 10.1002/smll.200700199. 17768776

[fsn370217-bib-0051] Li, P. R. , D. S. Zhang , T. B. Su , et al. 2020. “Genome‐Wide Analysis of mRNA and lncRNA Expression and Mitochondrial Genome Sequencing Provide Insights Into the Mechanisms Underlying a Novel Cytoplasmic Male Sterility System, BVRC‐CMS96, in *Brassica rapa* .” Theoretical and Applied Genetics 133, no. 7: 2157–2170. 10.1007/s00122-020-03587-z. 32399654

[fsn370217-bib-0052] Li, S. , D. Zhang , J. Wu , et al. 2015. “Purification, Preliminary Characterization and Bioactivities of Polysaccharides From Ostrea Rivularis Gould.” International Journal of Biological Macromolecules 80: 16–22. 10.1016/j.ijbiomac.2015.06.024. 26093318

[fsn370217-bib-0053] Li, X. H. , S. G. Li , G. P. Shi , et al. 2022. “Quantitative Proteomics Insights Into Gel Properties Changes of Myofibrillar Protein From *Procambarus clarkii* Under Cold Stress.” Food Chemistry 372: 130935. 10.1016/j.foodchem.2021.130935. 34818725

[fsn370217-bib-0054] Liang, Q. , S. Yu , S. Liu , et al. 2015. “Antibacterial Activity of the Coelomic Fluid From a Marine Worm, *Phasolosma esculenta* .” Journal of Marine Sciences 33, no. 2: 70–75. 10.3969/j.issn.1001-909X.2015.02.010.

[fsn370217-bib-0055] Liang, R. 2008. “Orthogonal Test Design for Optimization of the Extraction of Polysaccharides From *Phascolosoma esulenta* and Evaluation of Its Immunity Activity.” Carbohydrate Polymmers 73, no. 4: 558–563. 10.1016/j.carbpol.2007.12.026. 26048221

[fsn370217-bib-0056] Lin, B. , S. Wang , A. Zhou , Q. Hu , and G. Huang . 2023. “Ultrasound‐Assisted Enzyme Extraction and Properties of Shatian Pomelo Peel Polysaccharide.” Ultrasonics Sonochemistry 98: 106507. 10.1016/j.ultsonch.2023.106507. 37406540 PMC10422119

[fsn370217-bib-0057] Liu, L. , J. Cao , J. Chen , X. Zhang , Z. Wu , and H. Xiang . 2016. “Effects of Peptides From *Phascolosoma esculenta* on Spatial Learning and Memory via Anti‐Oxidative Character in Mice.” Neuroscience Letters 631: 30–35. 10.1016/j.neulet.2016.08.012. 27519930

[fsn370217-bib-0058] Liu, R. , M. Wang , J. Duan , J. Guo , and Y. Tang . 2010. “Purification and Identification of Three Novel Antioxidant Peptides From Cornu Bubali (Water Buffalo Horn).” Peptides 31, no. 5: 786–793. 10.1016/j.peptides.2010.02.016. 20206218

[fsn370217-bib-0059] Liu, Y. , C. Du , C. Lin , X. Gao , J. Zhu , and C. Zhang . 2022. “Characterization of Copper/Zinc Superoxide Dismutase Activity on *Phascolosoma esculenta* (Sipuncula: Phascolosomatidea) and Its Protection From Oxidative Stress Induced by Cadmium.” International Journal of Molecular Sciences 23, no. 20: 12136. 10.3390/ijms232012136. 36292990 PMC9602484

[fsn370217-bib-0060] Long, L. , Z. Sheng , and J. Zhu . 2015. “Ultrastructural Observations on Spermiogenesis in the Peanut Worm, *Phascolosoma esculenta* (Sipuncula: Phascolosomatidea).” Animal Cells and Systems 19, no. 3: 221–229. 10.1080/19768354.2015.1026398.

[fsn370217-bib-0061] Lu, J. , and A. Holmgren . 2014. “The Thioredoxin Antioxidant System.” Free Radical Biology and Medicine 66: 75–87. 10.1016/j.freeradbiomed.2013.07.036. 23899494

[fsn370217-bib-0062] Lutz, S. 2010. “Beyond Directed Evolution‐Semi‐Rational Protein Engineering and Design.” Current Opinion in Biotechnology 21, no. 6: 734–743. 10.1016/j.copbio.2010.08.011. 20869867 PMC2982887

[fsn370217-bib-0063] Matak, K. E. , R. Tahergorabi , and J. Jaczynski . 2015. “A Review: Protein Isolates Recovered by Isoelectric Solubilization/Precipitation Processing From Muscle Food By‐Products as a Component of Nutraceutical Foods.” Food Research International 77: 697–703. 10.1016/j.foodres.2015.05.048.

[fsn370217-bib-0064] McCord, J. M. , and I. Fridovich . 1969. “Superoxide Dismutase. An Enzymic Function for Erythrocuprein (Hemocuprein).” Journal of Biological Chemistry 244, no. 22: 6049–6055. 5389100

[fsn370217-bib-0065] Méndez, L. , L. G. Fidalgo , M. Pazos , et al. 2017. “Lipid and Protein Changes Related to Quality Loss in Frozen Sardine (*Sardina pilchardus*) Previously Processed Under High‐Pressure Conditions.” Food and Bioprocess Technology 10, no. 2: 296–306. 10.1007/s11947-016-1815-x.

[fsn370217-bib-0066] Meng, J. , X. Gao , S. Luo , et al. 2021. “Cloning, Functional Characterization and Response to Cadmium Stress of the Thioredoxin‐Like Protein 1 Gene From *Phascolosoma esculenta* .” International Journal of Moleular Sciences 23, no. 1: 332. 10.3390/ijms23010332. PMC8745482 35008758

[fsn370217-bib-0067] Meng, J. , G. Zhang , and W. Wang . 2022. “Functional Heterogeneity of Immune Defenses in Molluscan Oysters *Crassostrea hongkongensis* Revealed by High‐Throughput Single‐Cell Transcriptome.” Fish & Shellfish Immunology 120: 202–213. 10.1016/j.fsi.2021.11.027. 34843943

[fsn370217-bib-0068] Miller, T. E. , J. Dodd , D. J. Ormrod , and R. Geddes . 1993. “Anti‐Inflammatory Activity of Glycogen Extracted From Perna Canaliculus (NZ Green‐Lipped Mussel).” Agents and Actions 38: C139–C142. 10.1007/BF01991164. 8317309

[fsn370217-bib-0069] Ming, T. , H. Huan , C. Su , et al. 2021. “Structural Comparison of Two Ferritins From the Marine Invertebrate *Phascolosoma esculenta* .” FEBS Open Bio 11, no. 3: 793–803. 10.1002/2211-5463.13080. PMC7931202 33448656

[fsn370217-bib-0070] Ming, T. , Y. Wu , H. Huan , et al. 2021. “Integrative Proteomics and Metabolomics Profiling of the Protective Effects of Phascolosoma Esculent Ferritin on BMSCs in cd(II) Injury.” Ecotoxicology and Environmental Safety 212: 111995. 10.1016/j.ecoenv.2021.111995. 33529923

[fsn370217-bib-0071] Mou, X. , A. Zhang , H. Tao , et al. 2023. “Organoid Models for Chinese Herbal Medicine Studies.” Acta Materia Médica 2, no. 1: 64–71. 10.15212/AMM-2022-0047.

[fsn370217-bib-0072] Mourão, P. A. S. , M. S. Pereira , M. S. G. Pavão , et al. 1996. “Structure and Anticoagulant Activity of a Fucosylated Chondroitin Sulfate From Echinoderm: Sulfated Fucose Branches on the Polysaccharide Accout for Its High Anticoagulant Action.” Jouornal of Biological Chemistry 271, no. 39: 23973–23984. 10.1074/jbc.271.39.23973. 8798631

[fsn370217-bib-0073] Niu, R. L. , and J. H. Tang . 2012. “Research of Anti‐Fatigue Effect of *Phascolosoma esculenta* .” Science and Technology of Food Industry 33, no. 24: 389–391. 10.13386/j.issn1002-0306.2012.24.006.

[fsn370217-bib-0074] Niu, R. L. , Y. N. Xiong , and J. M. Y. Lu . 2011. “Study on Fibrinolytic Enzyme From *Phascolosoma esculenta* .” Chinese Journal of Marine Drugs 30, no. 6: 31–35. 10.13400/j.cnki.cjmd.2011.06.001.

[fsn370217-bib-0075] Parge, H. E. , R. A. Hallewell , and J. A. Tainer . 1992. “Atomic Structures of Wild‐Type and Thermostable Mutant Recombinant Human Cu, Zn Superoxide Dismutase.” Proceedings of the National Academy of Sciences of the United States of America 89, no. 13: 6109–6113. 10.1073/pnas.89.13.6109. 1463506 PMC49447

[fsn370217-bib-0076] Piepho, R. W. 2000. “Overview of the Angiotensin‐Converting‐Enzyme Inhibitors.” American Journal of Health‐System Pharmacy 57: S3–S7. 10.1093/ajhp/57.suppl_1.S3. 11030016

[fsn370217-bib-0077] Qu, H. , Y. Wu , Z. Luo , Q. Dong , H. Yang , and C. Dai . 2023. “An Efficient Approach for Extraction of Polysaccharide From Abalone (*Haliotis Discus Hannai* Ino) Viscera by Natural Deep Eutectic Solvent.” International Journal of Biological Macromolecules 244: 125336. 10.1016/j.ijbiomac.2023.125336. 37327933

[fsn370217-bib-0078] Ramya, R. , B. M. Subramanian , V. Sivakumar , R. L. Senthilkumar , K. Rao , and V. A. Srinivasan . 2011. “Expression and Solubilization of Insect Cell‐Based Rabies Virus Glycoprotein and Assessment of Its Immunogenicity and Protective Efficacy in Mice.” Clinical and Vaccine Immunology 18, no. 10: 1673–1679. 10.1128/cvi.05258-11. 21813661 PMC3187018

[fsn370217-bib-0079] Rivera‐de‐Torre, E. , C. Rimbault , T. P. Jenkins , et al. 2022. “Strategies for Heterologous Expression, Synthesis, and Purification of Animal Venom Toxins.” Frontiers in Bioengineering and Biotechnology 9: 811905. 10.3389/fbioe.2021.811905. 35127675 PMC8811309

[fsn370217-bib-0080] Saxena, A. , B. P. Tripathi , M. Kumar , and V. K. Shahi . 2009. “Membrane‐Based Techniques for the Separation and Purification of Proteins: An Overview.” Advances in Colloid and Interface Science 145, no. 1–2: 1–22. 10.1016/j.cis.2008.07.004. 18774120

[fsn370217-bib-0081] Schutgens, F. , and H. Clevers . 2020. “Human Organoids: Tools for Understanding Biology and Treating Diseases.” Annual Review of Pathology: Mechanisms of Disease 15: 211–234. 10.1146/annurev-pathmechdis-012419-032611. 31550983

[fsn370217-bib-0082] Schutz, A. , F. Bernhard , N. Berrow , et al. 2023. “A Concise Guide to Choosing Suitable Gene Expression Systems for Recombinant Protein Production.” STAR Protocols 4, no. 4: 102572. 10.1016/j.xpro.2023.102572. 37917580 PMC10643540

[fsn370217-bib-0083] Shahidi, F. , and P. Ambigaipalan . 2018. “Omega‐3 Polyunsaturated Fatty Acids and Their Health Benefits.” Annual Review of Food Science and Technology 9: 345–381. 10.1146/annurev-food-111317-095850. 29350557

[fsn370217-bib-0084] Shavandi, A. , Z. Hu , S. S. Teh , et al. 2017. “Antioxidant and Functional Properties of Protein Hydrolysates Obtained From Squid Pen Chitosan Extraction Effluent.” Food Chemistry 227: 194–201. 10.1016/j.foodchem.2017.01.099. 28274422

[fsn370217-bib-0085] Shen, W. , C. Liu , J. Ni , et al. 2021. “Effects of Low Temperature Stress on the Morphology and hsp70 and hsp90 Gene Expression of *Phascolosoma esculenta* .” Journal of Ocean University of China 20, no. 1: 159–168. 10.1007/s11802-021-4475-z.

[fsn370217-bib-0086] Shu, F. , T. Li , R. Zhou , D. Qian , and S. Zhou . 2023. “Antimicrobial Activity and Physicochemical Properties Analysis of Leukocyte Antibacterial Substances From *Phascolosoma esculenta* .” Journal of Biology 40, no. 3: 80–84. 10.3969/j.issn.2095-1736.2023.03.080.

[fsn370217-bib-0087] Su, X. , L. Du , Y. Li , et al. 2009. “Production of Recombinant Protein and Polyclonal Mouse Antiserum for Ferritin From Sipuncula *Phascolosoma esculenta* .” Fish & Shellfish Immunology 27, no. 3: 466–468. 10.1016/j.fsi.2009.06.014. 19563895

[fsn370217-bib-0088] Su, X. , L. Du , Y. Li , Y. Li , J. Zhou , and T. Li . 2010. “Cloning and Expression of HSP70 Gene of Sipuncula *Phascolosoma esculenta* .” Fish & Shellfish Immunology 28, no. 3: 461–466. 10.1016/j.fsi.2009.12.014. 20034571

[fsn370217-bib-0090] Traina, A. , G. Bono , M. Bonsignore , et al. 2019. “Heavy Metals Concentrations in Some Commercially Key Species From Sicilian Coasts (Mediterranean Sea): Potential Human Health Risk Estimation.” Ecotoxicology and Environmental Safety 168: 466–478. 10.1016/j.ecoenv.2018.10.056. 30419523

[fsn370217-bib-0091] Undeland, I. , S. D. Kelleher , and H. O. Hultin . 2002. “Recovery of Functional Proteins From Herring ( *Clupea harengus* ) Light Muscle by an Acid or Alkaline Solubilization Process.” Journal of Agricultural and Food Chemistry 50, no. 25: 7371–7379. 10.1021/jf020199u. 12452661

[fsn370217-bib-0092] Wang, C. Y. , H. W. Huang , C. P. Hsu , and B. B. Yang . 2016. “Recent Advances in Food Processing Using High Hydrostatic Pressure Technology.” Critical Reviews in Food Science and Nutrition 56, no. 4: 527–540. 10.1080/10408398.2012.745479. 25629307

[fsn370217-bib-0093] Wang, J. , P. Wang , Z. Zeng , et al. 2022. “Trabectedin in Cancers: Mechanisms and Clinical Applications.” Current Pharmaceutical Design 28, no. 24: 1949–1965. 10.2174/1381612828666220526125806. 35619256

[fsn370217-bib-0094] Wang, M. , Y. Li , X. Su , et al. 2010. “Construction and Analysis of a cDNA Library for *Phascolosoma esculenta* .” Journal of Applied Oceanography 29, no. 1: 20–26. 10.3969/J.ISSN.1000-8160.2010.01.004.

[fsn370217-bib-0095] Wang, M. , X. Su , Y. Li , Z. Jun , and T. Li . 2010. “Cloning and Expression of the Mn‐SOD Gene From *Phascolosoma esculenta* .” Fish & Shellfish Immunology 29, no. 5: 759–764. 10.1016/j.fsi.2010.07.005. 20654721

[fsn370217-bib-0096] Wang, W. , L. Zhang , Z. Wang , X. Wang , and Y. Liu . 2019. “Physicochemical and Sensory Variables of Maillard Reaction Products Obtained From *Takifugu obscurus* Muscle Hydrolysates.” Food Chemistry 290: 40–46. 10.1016/j.foodchem.2019.03.065. 31000054

[fsn370217-bib-0097] Wang, Y. , P. Xue , M. Cao , T. Yu , S. Lane , and H. Zhao . 2021. “Directed Evolution: Methodologies and Applications.” Chemical Reviews 121, no. 20: 12384–12444. 10.1021/acs.chemrev.1c00260. 34297541

[fsn370217-bib-0098] Wang, Y. , and J. Yu . 2021. “Membrane Separation Processes for Enrichment of Bovine and Caprine Milk Oligosaccharides From Dairy Byproducts.” Comprehensive Reviews in Food Science and Food Safety 20, no. 4: 3667–3689. 10.1111/1541-4337.12758. 33931948

[fsn370217-bib-0099] Wu, H. , Y. Gao , Z. Huang , and X. Jiang . 2015. “Enrichment of Cd2+ and Hg2+ in Phascolosoma Esculenta and Their Effects.” Chinese Journal of Applied Ecology 26, no. 6: 1871–1876. 10.13287/j.1001-9332.20150413.002. 26572044

[fsn370217-bib-0100] Wu, H. , Y. Liu , M. Guo , J. Xie , and X. Jiang . 2014. “A Virtual Screening Method for Inhibitory Peptides of Angiotensin I‐Converting Enzyme.” Journal of Food Science 79, no. 9: C1635–C1642. 10.1111/1750-3841.12559. 25154376

[fsn370217-bib-0101] Wu, Y. , M. Fang , L. Du , et al. 2014. “The Nutritional Composition and Anti‐Hypertensive Activity on Spontaneously Hypertensive Rats of Sipuncula *Phascolosoma esculenta* .” Food & Function 5, no. 9: 2317–2323. 10.1039/c4fo00416g. 25075455

[fsn370217-bib-0102] Wu, Y. , H. Jiang , J. Lin , J. Liu , C. Wu , and R. Xu . 2020. “Antioxidant, Hypolipidemic and Hepatic Protective Activities of Polysaccharides From *Phascolosoma esculenta* .” Marine Drugs 18, no. 3: 158. 10.3390/md18030158. 32178323 PMC7142949

[fsn370217-bib-0103] Xing, J. M. , J. Zou , X. D. Liu , et al. 2024. “Effects of Acute Hypoxia and Reoxygenation on the Coelomic Fluid of *Phascolosoma esculenta*: Oxidative Stress and Transcriptome Analysis.” Aquaculture Reports 39: 102424. 10.1016/j.aqrep.2024.102424.

[fsn370217-bib-0104] Xiong, Q. , Z. Song , W. Hu , et al. 2020. “Methods of Extraction, Separation, Purification, Structural Characterization for Polysaccharides From Aquatic Animals and Their Major Pharmacological Activities.” Critical Reviews in Food Science and Nutrition 60, no. 1: 48–63. 10.1080/10408398.2018.1512472. 30285473

[fsn370217-bib-0105] Yang, Z. , Y. Pan , J. Chen , et al. 2019. “Anti‐Inflammatory, Anti‐Oxidative Stress Effect of *Phascolosoma esculenta* Oligosaccharides on *Escherichia coli*‐Induced Sepsis Mice.” Food Science and Biotechnology 28, no. 6: 1871–1879. 10.1007/s10068-019-00620-w. 31807361 PMC6859150

[fsn370217-bib-0106] Ying, X. P. , H. U. Dahms , X. M. Liu , et al. 2009. “Development of Germ Cells and Reproductive Biology in the Sipunculid *Phascolosoma esculenta* .” Aquaculture Research 40, no. 3: 305–314. 10.1111/j.1365-2109.2008.02093.x.

[fsn370217-bib-0107] You, C. , L. Dong , M. Zeng , Y. Jiang , M. Xu , and Q. Zhang . 2019. “Effect of Acute Low‐Salinity Stress on the Survival and Na+/K+‐ATPase and Phosphatase Activities of *Phascolosoma esculenta* .” Marine Sciences 43, no. 3: 82–89. 10.11759/hykx20190117001.

[fsn370217-bib-0108] Zang, J. , H. Chen , G. Zhao , F. Wang , and F. Ren . 2017. “Ferritin Cage for Encapsulation and Delivery of Bioactive Nutrients: From Structure, Property to Applications.” Critical Reviews in Food Science and Nutrition 57, no. 17: 3673–3683. 10.1080/10408398.2016.1149690. 26980693

[fsn370217-bib-0109] Zhang, Y. , S. Chen , Y. Wang , et al. 2020. “Study on Antibacterial Properties of the Coelomic Fluid From *Phasolosma esculenta* .” Chinese Journal of Antibiotics 45, no. 8: 756–762. 10.13461/j.cnki.cja.006824.

[fsn370217-bib-0110] Zhang, Y. J. , Y. Han , M. Zhang , et al. 2022. “The Cotton Mitochondrial Chimeric Gene *orf610a* Causes Male Sterility by Disturbing the Dynamic Balance of ATP Synthesis and ROS Burst.” Crop Journal 10, no. 6: 1683–1694. 10.1016/j.cj.2022.02.008.

[fsn370217-bib-0111] Zheng, M. , Y. Jiang , C. You , X. Liu , and H. Liu . 2017. “Effects of Salinity and Body Weight on Oxygen Consumption and Ammonia Excretion of Sipuncula *Phascolosoma esculenta* .” Fisheries Science 36, no. 5: 647–651. 10.16378/j.cnki.1003-1111.2017.05.018.

[fsn370217-bib-0112] Zhong, S. , X. Ma , Y. Jiang , et al. 2022. “The Draft Genome of Chinese Endemic Species *Phascolosoma esculenta* (Sipuncula, Phascolosomatidae) Reveals the Phylogenetic Position of Sipuncula.” Frontiers in Genetics 13: 910344. 10.3389/fgene.2022.910344. 35937983 PMC9354978

[fsn370217-bib-0113] Zhou, F. , B. Cai , S. Ruan , and Q. Wei . 2024. “Purification, Characterization, and Antioxidant Ability of Polysaccharides From *Phascolosoma esculenta* s.” Food Science & Nutrition 12: 2799–2808. 10.1002/fsn3.3961. 38628168 PMC11016387

